# An integrated microfluidics platform with high-throughput single-cell cloning array and concentration gradient generator for efficient cancer drug effect screening

**DOI:** 10.1186/s40779-022-00409-9

**Published:** 2022-09-22

**Authors:** Biao Wang, Bang-Shun He, Xiao-Lan Ruan, Jiang Zhu, Rui Hu, Jie Wang, Ying Li, Yun-Huang Yang, Mai-Li Liu

**Affiliations:** 1 Key Laboratory of Magnetic Resonance in Biological Systems, State Key Laboratory of Magnetic Resonance and Atomic and Molecular Physics, National Center for Magnetic Resonance in Wuhan, Wuhan Institute of Physics and Mathematics, Innovation Academy for Precision Measurement Science and Technology-Wuhan National Laboratory for Optoelectronics, Chinese Academy of Sciences, Wuhan, 430071 China; 2 Department of Laboratory Medicine, Nanjing First Hospital, Nanjing Medical University, Nanjing, 210006 China; 3 Department of Hematology, Renmin Hospital, Wuhan University, Wuhan, 430060 China; 4 University of Chinese Academy of Sciences, Beijing, 10049 China; 5 Canary Center at Stanford for Cancer Early Detection, Department of Radiology, School of Medicine, Stanford University, Palo Alto, CA 94304 USA

**Keywords:** Microfluidics, Single-cell analysis, Leukemia, High-throughput drug screening, Single-cell cloning

## Abstract

**Background:**

Tumor cell heterogeneity mediated drug resistance has been recognized as the stumbling block of cancer treatment. Elucidating the cytotoxicity of anticancer drugs at single-cell level in a high-throughput way is thus of great value for developing precision therapy. However, current techniques suffer from limitations in dynamically characterizing the responses of thousands of single cells or cell clones presented to multiple drug conditions.

**Methods:**

We developed a new microfluidics-based “SMART” platform that is Simple to operate, able to generate a Massive single-cell array and Multiplex drug concentrations, capable of keeping cells Alive, Retainable and Trackable in the microchambers. These features are achieved by integrating a Microfluidic chamber Array (4320 units) and a six-Concentration gradient generator (MAC), which enables highly efficient analysis of leukemia drug effects on single cells and cell clones in a high-throughput way.

**Results:**

A simple procedure produces 6 on-chip drug gradients to treat more than 3000 single cells or single-cell derived clones and thus allows an efficient and precise analysis of cell heterogeneity. The statistic results reveal that Imatinib (Ima) and Resveratrol (Res) combination treatment on single cells or clones is much more efficient than Ima or Res single drug treatment, indicated by the markedly reduced half maximal inhibitory concentration (IC_50_). Additionally, single-cell derived clones demonstrate a higher IC_50_ in each drug treatment compared to single cells. Moreover, primary cells isolated from two leukemia patients are also found with apparent heterogeneity upon drug treatment on MAC.

**Conclusion:**

This microfluidics-based “SMART” platform allows high-throughput single-cell capture and culture, dynamic drug-gradient treatment and cell response monitoring, which represents a new approach to efficiently investigate anticancer drug effects and should benefit drug discovery for leukemia and other cancers.

**Supplementary Information:**

The online version contains supplementary material available at 10.1186/s40779-022-00409-9.

## Background

Although the overall death rate of leukemia has decreased slightly in recent years, drug resistance (DR) or DR-induced disease relapse is still the main cause of leukemia treatment failure [1, 2]. It is estimated that there were approximately 61,090 new leukemia diagnoses with about 23,660 deaths in the USA in 2021, according to a report of the National Cancer Institute. There is increasing evidence showing that DR is closely associated with leukemia cell heterogeneity, allowing some leukemic cells (e.g., leukemia stem cells) to survive after drug treatment, leading to disease recurrence [3, 4]. Traditional methods rely on sophisticated instruments to manually isolate small numbers of cells of interest, with individual analysis taking weeks to months, which is inefficient in view of large numbers of samples and patient variations [5]. Techniques that use single-cell analysis, such as flow cytometry, have had a remarkable effect on facilitating the analysis of genomic and transcriptomic heterogeneity in an end-point manner [6]. However, to date, the dynamic characterization of single cell profiles (e.g., morphology, proliferation, and cytotoxicity) using high-throughput techniques remains a significant challenge, hindering our understanding of the physiological and pathological progress of leukemia [7].

Microfluidics, particularly microfluidic chip technology, represents an attractive alternative for single cell studies [8, 9]. The microscale channel or chamber inside the chips has similar dimensions to those of most cells, allowing excellent performance in single-cell manipulations and guaranteeing the precision of single-cell analysis [10]. Furthermore, the use of multiple parallel micro-units (e.g., microtraps, droplets, and micropatterns) allows high-throughput single-cell analysis [11, 12]. Notably, the feasibility of integration with functional on-chip modules (e.g., a chemical gradient generator) or surrounding equipment (e.g., microscope, and mass spectrometer) enables the construction of a microenvironment close to physiological conditions and the application of dynamic spatiotemporal analysis [13]. Thus, microfluidics has been widely used in the field of single-cell analysis [8].

Owing to the above-mentioned advantages, several different types of microfluidic strategies including hydrodynamic and droplet platforms have been developed for single cell DR analysis [14–16]. Compared with other approaches, hydrodynamic-based microfluidics is a simple but efficient way to generate a high-throughput single-cell array for cytotoxicity testing [17]. For example, Pang et al. [18, 19] demonstrated this advantage by employing microchannels and microchambers with different sizes to construct single tumor cell and single-cell derived spheroid array for the exploration of the relationship between cell deformability and its resistance to anticancer drugs. Yellen et al. [7] established a microchamber array with approximately 6000 units and introduced automatically imaging process to analyze single cell phenotypic heterogeneity upon drug treatment. They conducted an eight-chip study of MOLM-13 cells [an acute myeloid leukemia (AML) cell line] exposed to different concentrations of the FLT3 inhibitor quizartinib to investigate the drug effects on cell growth. These studies demonstrated the capability of microfluidic devices for massive single cell DR analysis, although the architecture of the device was relatively complex and only one drug condition could be tested on each chip.

As the development of DR in cancer cells depends significantly on the drug concentrations, it is of vital importance to study the performance of single cells under different drug gradients [20, 21]. However, off-chip drug gradient generation is often labor consuming, and could lead to variations in other conditions besides drug concentration across different chips, which is not ideal for efficient DR analysis. With regard to this, Pei et al. [22] combined a concentration gradient generator (CGG) with single cell array to explore the bioeffects of an anticancer drug on single circulating tumor cells collected from cancer patient samples. By integrating high frequency acoustic waves in a concentration gradient microfluidic device, Zhao et al. [23] developed a single-cell drug screening acceleration method for the evaluation of AML chemotherapy. While these studies illustrate the feasibility of on-chip CGG to facilitate drug-screening studies, these proposed platforms could only handle several hundreds of single cells and lacked the capability for long-term cell retention and on-chip cell culture. Taken together, current technologies have shown the feasibility and advantages of microfluidic strategies for single cell DR analysis, but a SMART (Simple, Massive and Multiplex, Alive, Retainable, and Trackable) platform that is easy to control and allows high-throughput single-cell capture, culture, and dynamic imaging for efficient drug screening is still lacking.

To develop a new DR analysis platform that can be used for high-throughput and dynamic characterization of single cells and single-cell derived clones, we proposed a fully integrated microfluidic device with a 6-channel CGG and a 4320-microchamber array. The CGG was constructed based on the classical Christmas tree-shaped network to provide 6 drug-concentration gradients simultaneously. The cell-trapping array, located downstream of the CGG module, was carefully designed and arranged to allow high-throughput and high-efficiency single-cell capture, cloning, and identification. Cells of the leukemia cell line K562 were comprehensively characterized on the device after the treatment of two well-known drugs, Imatinib (Ima) and Resveratrol (Res). Additionally, cells collected from clinical AML patient samples were also investigated with treatment by Daunorubicin (DNR) and Cytarabine (Ara-C).

## Methods

### Cell culture

Human chronic myeloid leukemia (CML) cell line (K562) was purchased from ATCC (CCL-243; Manassas, VA, USA), and maintained in RPMI 1640 medium (22400089; Gibco, Grand Island, NY, USA) supplemented with 10% (v/v) fetal bovine serum (FBS; 10099-141; Gibco, Grand Island, NY, USA) and 1% (v/v) penicillin–streptomycin (P1400; Solarbio, Beijing, China) in a humidified atmosphere of 5% CO_2_ at 37 °C. K562 cells grow and proliferate in a suspended state. Cell density in culture flasks was controlled below 1.0 × 10^6^ cells/ml, and the cells were passaged every 2–3 d to ensure that they were in an exponential growth phase.

### Device design and fabrication

The device used in this study [containing a Microfluidic chamber Array (4320 units) and a six-Concentration gradient generator (MAC)] consists of a top polydimethylsiloxane (PDMS) layer containing the micropattern and a bottom glass slide. The micropattern was designed with Computer Assistant Design (CAD) software, and optimized with Computational Fluid Dynamics (CFD) simulation, shown in Additional file 1: Fig. S1. The optimal design was printed out as glass photomask to generate the microstructure mold fabricated following protocols similar to our previous reports [10, 24]. Briefly, negative photoresist (SU-8 3025; MicroChem Corp., MA, USA) was spin-coated onto a 4-inch silicon wafer at 3700 rpm for 1 min to form an 18-μm layer. After baking at 65 °C for 5 min and then 95 °C for 20 min, the wafer was cooled and exposed to UV light with the photomask for 6 s (5 mJ/cm^2^), followed by baking at 65 °C for 2 min and then at 95 °C for 5 min. Finally, the wafer was developed and heated at 135 °C for 60 min. After that, a 10:1 (w/w) mixture of PDMS and curing agent (Sylgard 184, Dow Corning, MI, USA) was poured onto the mold with a thickness of about 4 mm and heated at 65 °C for 2 h. Next, the patterned PDMS sheet, as shown in Additional file 1: Fig. S2a, was cut, peeled off, and punched to produce 1-mm diameter inlet and outlet. After ethanol rinsing, air blowing, and oxygen plasma treatment, the PDMS sheet and a glass slide were bonded together to form the microfluidic device.

### Device operation and characterization

Serial procedures, including single cell loading, culture, dye labeling of cells, chemical gradient generation and imaging, were tested on MAC to verify its ability and robustness. The devices were degassed by vacuum for approximately 10 min before use, which avoids the generation of bubble during sample loading. Next, 75% ethanol and sterile phosphate buffer saline (PBS) were sequentially introduced into the device for sterilization. Typically, cell suspensions with a density of 3 × 10^6^ cells/ml were used for cell loading. To examine cell capture efficiency of the device with limited cells, K562 cells were diluted to different densities (1.0 × 10^4^, 1.0 × 10^5^, and 1.0 × 10^6^ cells/ml) and tested. Cell suspensions were transferred into syringes adapted to syringe pump (KDS200; KD Scientific, MA, USA). Flexible tube (AAD04119; Tygon, PA, USA) was used to connect the syringes with the microfluidic device through stainless-steel needles. Cell suspension was then pumped into the device with a flow rate of 4 μl/min to capture single cells at the trap of microchambers (within 2 min). Then, the cell suspension was replaced with fresh culture medium. With an increased flow rate of 20 μl/min, the trapped single cells were docked into the microchambers (within 30 s). The microfluidic device was then transferred into an incubator and recombined to syringes containing culture medium at a position 10-cm higher than the device to drive medium flowing through the device based on the gravity difference to feed cells. During cell culture, medium was replaced on time and cell morphology was recorded with an automatic imaging system (EVOS, Thermo Fisher Scientific, MA, USA) at certain time points. For cell labeling, corresponding dyes, such as live/dead stains, were introduced into the device with syringe pump at a flow rate of 4 μl/min. After the staining, fresh medium was used to wash away the residual staining dyes and images at specific areas or across the whole device were acquired with EVOS.

### Characterization of on-chip chemical gradient generation

Concentration gradient generation on MAC was characterized by fluorescent dye mixing assay. K562 cells were first loaded, cultured and stained with Hoechst 33342 (H3570; Thermo Fisher Scientific, MA, USA;) on the device before the mixing assay, which is to closely mimic a real experiment employing drug gradients to treat the docked or cloned cells. In short, 5 µmol/L fluorescein sodium (F6377; Sigma-Aldrich, MO, USA) and sulforhodamine B (S1307; Thermo Fisher Scientific, MA, USA) dissolved in culture medium were introduced into the device with a syringe pump at different flow rates to find out the optimal rate that could generate a linear concentration gradient in the device. Additionally, a prolonged duration of up to 24 h was also recorded under the optimal mixing flow rate to test the stability of the chemical concentration pattern. Fluorescent images were taken with EVOS and processed with ImageJ software (National Institutes of Health) to quantify the chemical concentrations.

### Single or combined drug treatment on single K562 cells or single K562 cell-derived clones

Ima (T6230; TargetMol, MA, USA), a selective inhibitor of the BCR-ABL tyrosine kinase and the frontline drug for CML treatment, was used as the model drug for K562 cell DR analysis on our device [25]. Additionally, combined chemotherapy usually shows better outcome than single chemical treatment. Hence, we also combined Res (T1558; TargetMol, MA, USA), a natural extract from grapes, peanuts and others, with Ima to examine the potential of our device for combined chemotherapy [26]. Single and combined drug treatments were carried out on K562 single cells and single-cell derived clones on the device. Appropriate concentrations of the two drugs were first evaluated on the device based on preliminary experiments performed with cells cultured in Petri dishes. For single K562 cell cytotoxicity assay, single cell array was first generated and imaged on the device as mentioned above. Then medium containing Ima (3 μmol/L) or Res (300 μmol/L) or combined of these two drugs (Ima/Res, 2/150 μmol/L, the two concentrations were intendedly decreased in consideration of the relatively high cytotoxicity of the combined drugs shown in the preliminary experiments) were pumped into the device from one inlet at 0.05 μl/min for 24 h, while the other inlet was kept flowing culture medium. After that, sterile PBS was introduced into the device to wash away the drugs, followed by flowing fluorescent dyes of calcein-AM (C3099; Thermo Scientific, MA, USA) and propidium iodide (PI; P1304MP; Thermo Scientific, MA, USA) to stain the live and dead cells, respectively. Finally, fluorescent images were taken with EVOS for statistical analysis. Cytotoxicity assays on single K562 cell derived clones were performed similar to that of K562 single cells, except that the arrayed single cells were first cultured for 48 h to form clones on the device and the drug concentrations were correspondingly adjusted (Ima, 14 μmol/L; or Res, 500 μmol/L; or Ima/Res, 8/250 μmol/L).

### Clinical leukemia patient sample collection and cell preparation

To test the feasibility of our device for clinical application, patient-derived leukemia cell samples were tested in this work. We collected two deidentified acute myeloid leukemia (AML) patient samples. AML samples were selected because this type of leukemia shows more serious clinical symptoms than other types [3]. This study was approved by the Committee on Human Research of Renmin Hospital of Wuhan University (WDRY2022-K021). Anticoagulant bone marrow specimens were obtained from surgery at Renmin Hospital of Wuhan University and delivered following standard protocol. Density gradient centrifugation was first performed with Ficoll-Paque gradient (17-1440-02; GE, USA) to isolate mononuclear cells from the specimens under instruction. After that, anti-CD34 antibody-labelled magnetic microbeads (130-046-702; Miltenyi Biotec, Bergisch Gladbach, Germany) were used to purify the CD34-positive cells (CD34 is often used as a typical marker for AML cell isolation [27]) according to the manufacturer’s recommendations. The obtained cells (1.0 × 10^6^ cells for each sample) were cultured with RPMI 1640 medium for 2 h and then loaded into the device for drug test.

### On-chip drug treatment on single cells isolated from AML patients

Ara-C (T1272; TargetMol, MA, USA) and DNR (T1511; TargetMol, MA, USA), as the standard combined chemotherapy for AML, were employed in the assays [28]. Meanwhile, the size of the microchamber for cell trapping and retention was carefully adjusted to accommodate primary leukemia cells that are smaller than K562 cells. Specifically, the entrance of the microchamber was narrowed from 5.5 to 3.8 μm, and the inner area of the microchamber was compacted from 40 × 60 to 40 × 20 μm^2^. Ara-C and DNR were introduced into MAC with a combined ratio of 5:1 (10/2 µmol/L) for 24 h[29]. Drug-induced cell apoptosis analysis and data acquisition were processed as that of K562 cells.

### Image acquisition, processing and statistical analysis

Bright and fluorescent images were acquired with EVOS auto imaging system, which enables the capture of single image at a certain position or multiple images for a large area followed a stitching process to combine these images. Cell capture rate and single-cell capture rate were defined as the ratio of microchamber numbers with ≥ 1 cell or single cells to total microchambers, respectively. Cell size was determined with the “Analyze Particles” module of ImageJ software (National Institutes of Health), and a threshold was employed to eliminate non-target particles. Fluorescence intensity of the images in concentration gradient characterization was quantified with ImageJ software.

Cell viability is calculated based on the ratio of live cells (dyed green with calcein-AM) to the sum of live and dead cells (dyed red with PI). Cell proliferation rate in certain duration is defined as the ratio of live cells at the end time point to that at the start time point. As some cells lysed after drug stimulation, cell proliferation rate was used to evaluate on-chip drug effects. Additionally, microchambers involved in data extraction and analysis were selected evenly across the whole device, shown in Additional file 1: Fig. S2b. IC_50_ in each group of drug treatment experiments was calculated with Origin Basic Functions: Logistic dose response in Pharmaceutical/Chemistry. Data statistical analysis was carried out with Origin2019.

## Results

### Working principle of MAC

**Fig. 1:**
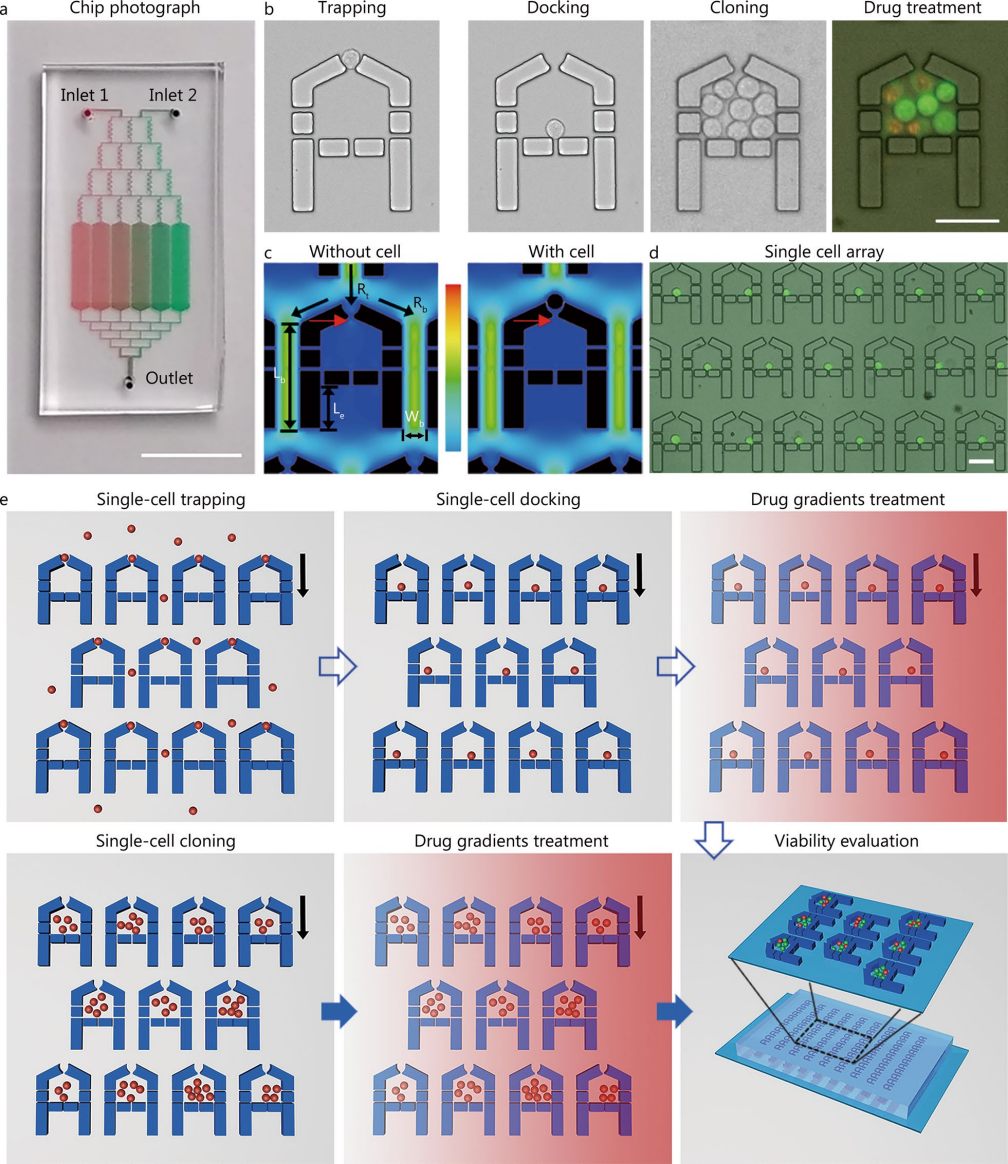
Design and working principle of the microfluidic device. **a** Photograph of a fabricated device filled with red and green dyes. Scale bar = 1 cm.** b** Micrographs showing the process of single cell trapping, docking, cloning and drug stimulation followed live/dead cell staining. Scale bar = 50 μm. **c** Simulation of the flow velocity distribution around the microchamber without or with a cell trapped. The red arrows indicate the differences of the flow rates. **d** Show case of captured single K562 cell array stained with calcein-AM. Scale bar = 50 μm. **e** Work flow of single cells or single-cell derived clones-based drug effect evaluation on the microfluidic device

We believe an efficient single-cell drug effect screening platform should have “SMART” features: S (Simple, the device fabrication and system setup are simple), M (Massive, the device allows the generation of thousands of massively arrayed single cells; Multiplex, the device itself can generate multiple drug concentration gradients with good linearity), A (Alive, the captured single cells have high viability, and can grow and proliferate), R (Retainable, the captured or proliferated cells can be well retained in the microchamber), and T (Trackable, it enables multiple time-point tracking for the arrayed cells and ease of locating the interested cells). Although various strategies have been proposed to meet these requirements, most have various limitations. Accordingly, we designed a SMART MAC for the analysis of single leukemia cell heterogeneity and drug susceptibility (Fig. 1a; Additional file 1: Figs. S1a, S2a). The microchannel of MAC is a one-layer architecture and consists of two inlets, a Christmas-tree like CGG, a cell-trapping array, and a ladder network connected to the outlet. The CGG can simultaneously produce 6 concentrations (Additional file 1: Fig. S1b), with each connected to a channel embedded with the single-cell trapping array. The device is fabricated using soft photolithography and rapid PDMS molding methods. It has a small footprint (approximately 30 × 20 mm^2^) that can be easily fitted into a culture dish and placed in a common cell culture incubator.

The trapping array includes 4320 (720 × 6) microchambers that are arranged in a mesh network with sequential numbers marked at selected positions to assist location, observation and analysis of the cells of interest (Additional file 1: Fig. S2a). The microchamber is designed like a “house” with a small entrance for single cell trapping and an expanded room (approximately 40 × 60 μm^2^) to enable single cell docking, culture, and stimulation (Fig. 1b). The entrance is designed as a “cup” that contains an upper opening constructed with two arcs with a 12-µm radius and a lower gap with approximately 5.5-µm width (Additional file 1: Fig. S1a), which is suitable for capturing cells with diameters ranging from 10 to 20 µm. The typical size of the microchamber (i.e., entrance and room) can be adjusted to accommodate different cells. Small gaps (2.8-µm width) are also present in the surrounding walls to guide fluid through the microchamber. The bypass between the microchambers, with a width W_b_ of 24 µm and a length L_b_ of 110 µm, is intended to transport solutions and uncaptured cells (Additional file 1: Fig. S1a). The specific design and dimensions of the microchamber were based on previous work and experience [24, 30] and should work well for most human cancer cells, although minor adjustments could be made to accommodate specific cells.

Single-cell capture in the microchamber is based on a self-limiting principle, in which the fluid profile is regulated by the presence of a cell at the opening or the ratio between the fluid resistance at the trap R_t_ and that at the bypass R_b_ (Fig. 1c, Additional file 1: Fig. S1a). R_b_ is positively correlated with L_b_ and negatively correlated with W_b_, and can be described as the following equation:

$${\text{R}} \approx \frac{{12{\upeta \text{L}}}}{{{\text{wh}}^{3} - 0.63{\text{h}}^{4} }},{\text{ w}} > {\text{h}},$$

where η is the dynamic viscosity of the fluid, L is the length of the channel, w and h represent the cross-sectional dimensions of the channel, in which the smaller of the two dimensions is defined as h. A prolonged L_b_ results in increase of R_b_, which facilitates single-cell capture at the top trap. However, longer L_b_ means lower density of microchambers or larger footprint of the device, which is inefficient to high-throughput single-cell analysis. Consequently, a balance should be established between single cell efficiency and throughput. Following this principle, we specifically elongated the length of the microchamber wall (L_e_, 45 µm), which significantly increased R_b_. When the opening is empty, cells tend to deterministically be captured in it; however, when the opening is occupied by a cell, R_t_ increases markedly and the fluid and other cells tend to flow through the bypass to fill the downstream blank traps. As expectedly, the simulation of the flow velocity distribution around the microchamber without or with the retention of a single cell in the trap agreed with the theoretical analysis (Fig. 1c, Additional file 1: Fig. S1c). After cell trapping and medium washing, the captured single cells can be efficiently docked into the microchambers (Fig. 1d) due to their inherit deformability. It should be noted that more than one cell (e.g., two cells adhered) could be captured in the trapping area and docked into the same chamber, although this possibility is very low due to the well-organized trap that only accommodates one cell. Typically, single cell loading and docking on the device can be completed within 5 min. Cells enclosed in the microchambers tend to remain in place despite fluid movement. With the continuous flow of the culture medium, the single cells proliferate, and single-cell derived clones can be formed in the microchambers. The single cells or clones can be treated with chemical gradients generated by the upstream CGG (Fig. 1e).

### MAC enables efficient single-cell capture and single-cell cloning

**Fig. 2:**
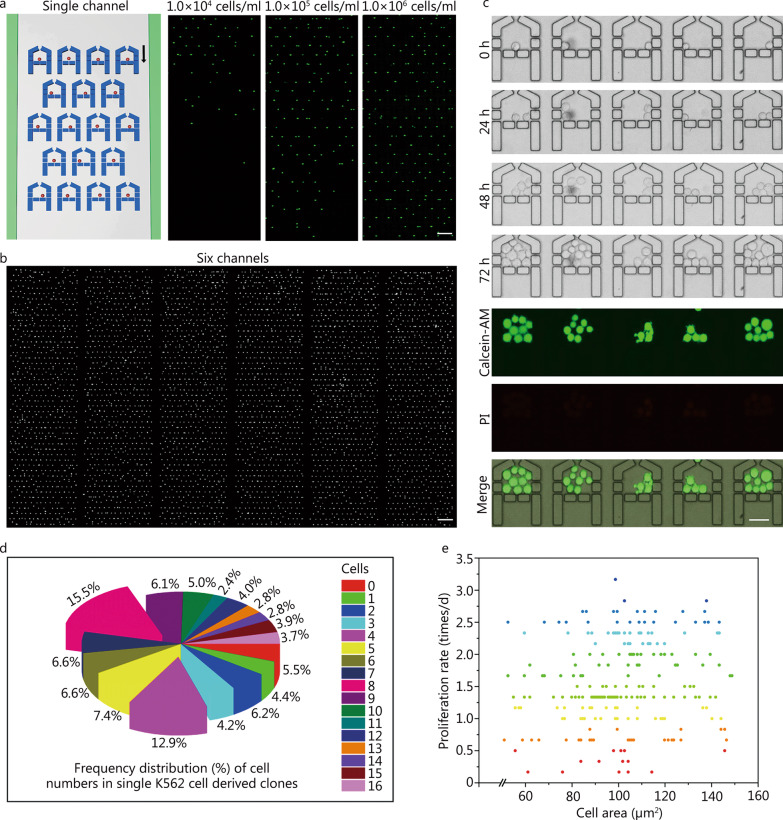
Evaluation of the efficiency of single-cell capture and single-cell cloning on the devices. **a** Capture rate in the single-plexed device with different cell densities. Scale bar = 200 μm. **b** Typical single-cell capturing micrograph showing the 4320 microchambers with about 3000 single cells captured in the array. Scale bar = 400 μm. **c** Time-lapse images of the same chambers showing single-cell capture, cloning and staining. Scale bar = 50 μm. **d** Statistics of the frequency distribution (%) of cell numbers in single K562 cell-derived clones’ experiment on MAC. The numbers indicate the cell numbers of different single-cell derived clones at t = 72 h. **e** Investigation of the relationship between cell division rate and initial cell size in some representative microchambers

Although many microfluidic methods have been reported, single-cell capture, retention, and culture still require improvement. An ideal micro-unit for dynamic single-cell characterization should simultaneously satisfy the requirements of highly efficient single-cell capture, sufficient space for single-cell proliferation, the capability of retaining the cells or clones, and ease in locating the target cells. Here, an array of house-like microchambers was designed to meet the above needs. Given that cell samples are often limited, cell capture efficiency (the number of microchambers occupied with cells to the total number of microchambers) under different cell densities (cells/ml) was first tested on a single-plex device (Fig. 2a, Additional file 1: Fig. S3a). Calcein-AM stained K562 cells were adjusted to different densities (1.0 × 10^4^, 1.0 × 10^5^, and 1.0 × 10^6^ cells/ml) and loaded onto different chips. As shown in Fig. 2a, most upstream microchambers were occupied by cells when the cell density was as low as 1.0 × 10^4^ cells/ml. The injected cells were preferentially captured close to the entrance and few cells were lost from the device, indicating the great performance of the design for capturing cells from samples with limited numbers of cells. Increased cell density resulted in an efficient occupation of the traps by single cells, reaching approximately 80% and 90% occupancy for 1.0 × 10^5^ and 1.0 × 10^6^ cells/ml, respectively (Fig. 2a). At the optimal cell density (3.0 × 10^6^ cells/ml), MAC with 6 channels showed an overall single-cell capture rate of 74% (*n* = 3), with a typical cell capturing image shown in Fig. 2b, implying that approximately 3000 single cells can be captured for drug testing in one single assay. In addition, almost all the single cells showed bright green fluorescence after docking, demonstrating high cell viability and minimal cell damage during the loading process.

Although cell heterogeneity can be revealed by single-cell analysis, deviations are inevitably present in individual cells. The expansion of single cells to single-cell derived clones for analysis can be an efficient strategy to eliminate such deviation [31]. With respect to this, single K562 cell derived clones were generated in our device for a more precise analysis of leukemic cell heterogeneity. Figure 2c shows the 72-h dynamic single-cell cloning process. About 90% of the initially captured single cells proliferate into single clones with varying cell numbers (Fig. 2d, Additional file 1: Fig. S3b), and the overall cell viability in the single clones was determined to be over 95% (Additional file 1: Fig. S4a). The variation in the numbers of cells within the clones demonstrated the remarkable heterogeneity of K562 cells. The average proliferation rate of single-cell derived clones in the device (Additional file 1: Fig. S4b) revealed the significant biocompatibility of our system. In addition, we analyzed the relationship between the single-cell proliferation rate and the initial cell size. Additional file 1: Fig. S4c demonstrates the accuracy of cell measurement using ImageJ-based methods. No apparent correlation was observed between the initial cell area and the proliferation rate (Fig. 2e), indicating that it is not sufficient to define K562 cell heterogeneity by only a single morphological feature, such as cell size. These results verified the feasibility of our device for high-throughput single cell and single-cell cloning analysis, which laid the foundation for the dynamic characterization of leukemic cell heterogeneity and the associated DR.

### Robust and simple chemical gradient generation

**Fig. 3:**
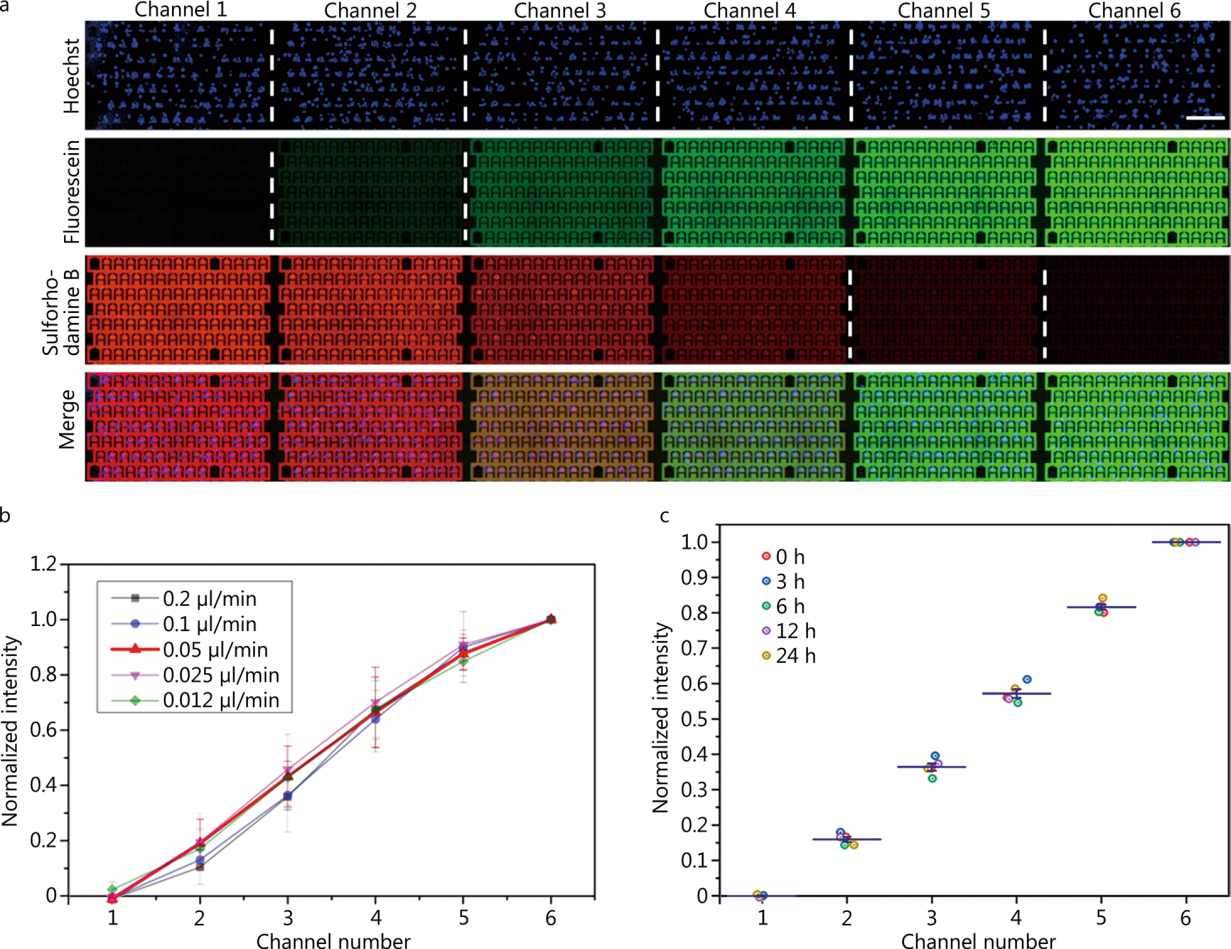
Characterization of concentration gradient generation on MAC with fluorescein sodium (green) and sulforhodamine B (red). **a** Representative area of the 6 channels showing the fluorescence distribution with single-cell derived clones (stained as Hoechst 33342) in the microchambers. Scale bar = 400 μm. **b** Normalized intensity of fluorescein under different flow rates in the 6 channels. **c** Normalized intensity of fluorescein at different time points under 0.05 μl/min in the 6 channels

Cell-based drug screening often requires the parallel evaluation of cytotoxicity with different drug concentrations, which is a technical challenge, especially for high-throughput screening (HTS). In this respect, the use of automatic handling by robots has been developed to enhance efficiency over manual operation. However, the high cost of such technology restricts its broad application. Owing to the intrinsic merits of flexible design and low sample consumption, microfluidics devices have been employed to produce desirable drug concentration regimes for drug HTS. The Christmas-tree like CGG is a simple and efficient microfluidic module to generate variable chemical gradients, and we employed it for the high-throughput analysis of leukemic single cell DR, with some minor adjustments. The CGG is designed in a Christmas-tree shape (Additional file 1: Fig. S2a) that includes mainly sine-shaped microchannels to improve the mixing efficiency using a longer distance and increased perturbance. Based on the principles of laminar flow and molecular diffusion, the input solutions are sequentially mixed and split; under optimal structural parameters and flow rates, the desired chemical gradient can be produced (Additional file 1: Fig. S1b). The performance of the CGG was evaluated by introducing fluorescein sodium (green) and sulforhodamine B (red) solutions into the device with single K562 cell derived clones (stained with a blue dye). Fluorescent images were taken to analyze the flow profile at different flow rates. It was observed that the fluorescent solutions were repeatedly mixed and split in the CGG network. Uniform streams with different concentrations were produced at the outputs and flowed into the downstream microchamber arrays (Additional file 1: Fig. S5a). Figure 3a shows a fraction of the 6 channels with a flow rate of 0.05 μl/min, showing the gradual distribution of the green and red fluorescence profiles, indicating the successful formation of a gradient of the two solutions and the lack of influence of the cells (stained as blue) in the microchambers. Additionally, varying the flow rate resulted in the distribution of different concentrations, with the formation of a linear gradient at 0.05 μl/min (Fig. 3b), which was employed in the subsequent assays. Furthermore, multiple monitoring at various time points demonstrated the stability of the concentration gradient with the flow rate of 0.05 μl/min (Fig. 3c, Additional file 1: Fig. S5b), demonstrating the capability of the system for long term drug stimulation. This concentration distribution was also used as the reference for drug concentration calculation in the following drug-based experiments (Additional file 1: Fig. S5). In conclusion, these experimental results on CGG performance corresponded well with the simulation results (Additional file 1: Fig. S1b), demonstrating the feasibility of our system for high-throughput DR analysis.

### K562 cells demonstrated significant heterogeneity in the cytotoxicity assay and various drug treatments resulted in different IC_50_ values

Cytotoxicity assays play key roles in drug screening and DR analysis. Here, the effects of Ima and Res on single K562 cells were characterized on MAC. The K562 cell line, established from a CML patient, harbors an abnormal fusion gene *BCR-ABL* encoding a tyrosine kinase, which promotes both cell proliferation and DR through several pathways [32, 33]. Ima is the first specific inhibitor of the BCR-ABL tyrosine kinase, and has revolutionized the treatment of CML, although DR is becoming a major problem [25]. In contrast to Ima, Res is a natural phytoalexin that has been shown to have nonspecific antioxidant and antitumor activities [26]. The combination of Ima and Res has been shown to improve the outcomes of leukemia therapy [34].

**Fig. 4:**
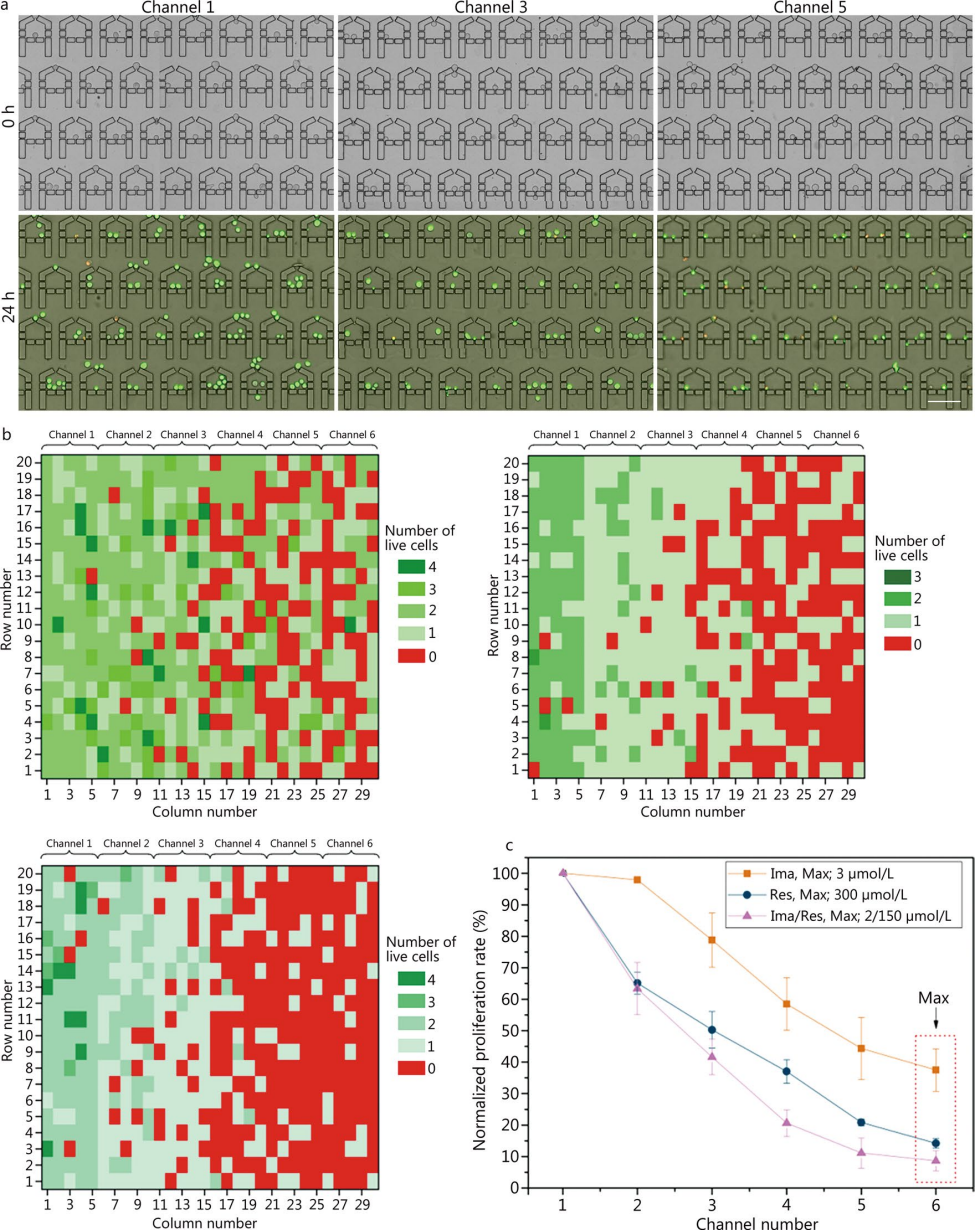
Single or combined drug treatment with Imatinib (Ima) and Resveratrol (Res) on single K562 cells captured in MAC. **a** Selected areas in the first (channel 1), third (channel 3) and fifth (channel 5) channel showing the single cells at t = 0 h and 24 h with the treatment of combined drugs Ima/Res. The drug concentrations in channel 1, 3 and 5 are 0, 0.84/63.09 and 1.68/126.06 μmol/L, respectively. After drug treatment, calcein-AM/PI were used to identify cell viability. Scale bar = 100 μm. **b** Live cells in the representative microchambers after Ima, Res and Ima/Res treatment. The drug concentrations in channels 1 − 6 are 0, 0.63, 1.26, 1.89, 2.52 and 3.00 μmol/L for Ima, and 0, 63.21, 126.18, 189.15, 252.12 and 300.00 μmol/L for Res, and 0, 0.42/31.61, 0.84/63.09, 1.26/94.58, 1.68/126.06 and 2/150 μmol/L for Ima/Res, respectively. **c** Overall cell proliferation rates under different drug treatments in the 6 channels with different drug concentrations. The sixth channel with the maximum drug concentration was indicated

A single-cell array was first generated on MAC. Then, Ima (3 μmol/L) or Res (300 μmol/L) or both (Ima/Res, 2/150 μmol/L) were used for on-chip experiments, using concentrations based on preliminary experiments. After drug stimulation for 24 h, the cells were stained with live/dead dyes for subsequent DR analysis. As expected, the number of live cells diminished as the drug concentrations increased (note that most of dead cells disintegrated to debris due to the drug cytotoxicity and flowed out through the gaps, leaving only a few red spots in the microchambers), and this negative correlation was consistent in all the three treatments (Fig. 4a, Additional file 1: Fig. S6). Quantification of the live cells across the whole device was also carried out, as shown in Fig. 4b, demonstrating not only the dose-dependent drug responses but also the heterogeneity of individual cells in response to the treatment. The uniform distribution of cell viability in each channel with the relevant drug concentration also demonstrates the presence of low system bias and the statistical independence of each cell. Notably, the surviving cells remaining in the channel 5 chambers may have been those that were tolerant to high drug concentrations. The results also suggested that cells in single-cell derived clones could respond differently to drug treatment. One of the possible reasons could be that these cells might be in different phases of the cell cycle with different sensitivities to the drugs [35]. The overall viability curves corresponding to the drug-treated single cells are shown in Fig. 4c. Combining the results of Fig. 4b–c and Additional file 1: Fig. S6, it can be seen that the combination of the two drugs resulted in higher cytotoxicity in the single cells compared with the single drugs, even though the drug concentrations were lowered intentionally in the combination group. The detailed information on the drug concentrations and the calculated IC_50_ values for each treatment are shown in Table 1. The three groups of Ima, Res and Ima/Res treatments on single K562 cell resulted in IC_50_ values of 1.57, 97.15 and 0.60/45.00 µmol/L, respectively. The relatively lower IC_50_ of Ima in comparison with Res indicates the stronger killing effect of Ima for leukemia treatment. At the same time, the IC_50_ of Ima/Res also demonstrated the advantage of using drug combinations over single drugs.

**Table 1:** Detailed drug concentrations and the calculated IC_50_ values of each treatment (drug concentration unit: µmol/L)

Treatments		Channel number						Calculated IC_50_
		1	2	3	4	5	6	
K562 cell line								
SC	Ima	0	0.63	1.26	1.89	2.52	3.00	1.57
	Res	0	63.21	126.18	189.15	252.12	300.00	97.15
	Ima/Res	0	0.42/31.61	0.84/63.09	1.26/94.58	1.68/126.06	2/150	0.60/45.00
SCDC	Ima	0	2.95	5.89	8.83	11.77	14.00	2.99
	Res	0	105.35	210.30	315.25	420.20	500.00	90.03
	Ima/Res	0	1.69/52.68	3.36/105.15	5.04/157.63	6.72/210.10	8/250	1.58/49.38
AML primary cell								
P1	Ara-C/DNR	0	2.11/0.42	4.21/0.84	6.31/1.26	8.40/1.68	10.0/2.0	1.75/0.35
P2	Ara-C/DNR	0	2.11/0.42	4.21/0.84	6.31/1.26	8.40/1.68	10.0/2.0	4.25/0.85

*SC* single cell, *SCDC* single-cell derived clones, *Ima* Imatinib, *Res* Resveratrol, *Ima/Res* Imatinib combined with Resveratrol, *AML* acute myeloid leukemia, *P1* number one patient, *P2* number two patient, *Ara-C* Cytarabine, *DNR* Daunorubicin, *IC*_*50*_ half maximal inhibitory concentration

### Single K562 cell-derived clones showed higher drug tolerance compared with single cells

**Fig. 5:**
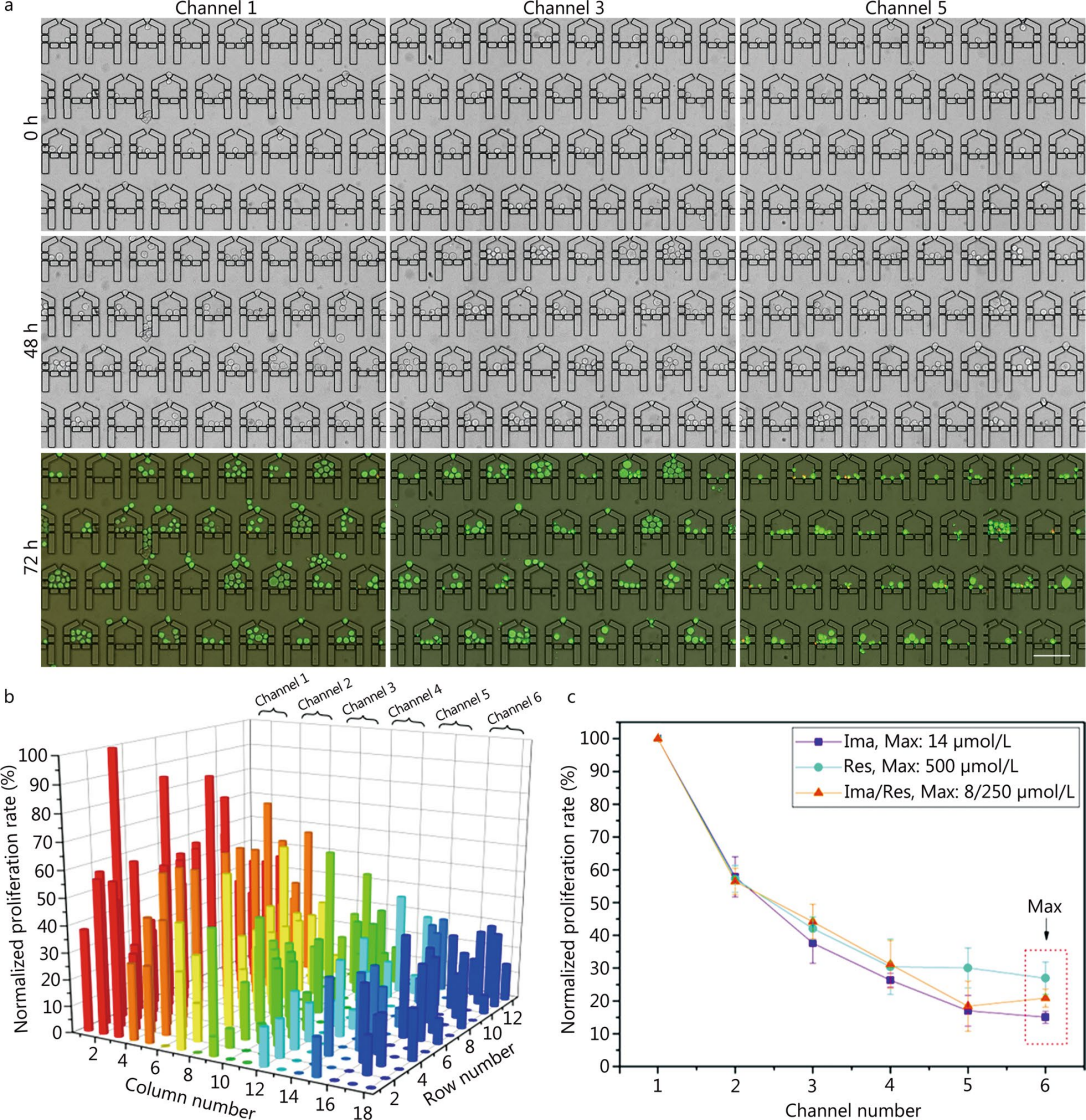
Combined drug treatment on single K562 cell-derived clones in MAC. **a** Representative area showing single cell captured (t = 0 h), cultured (t = 48 h) and Ima/Res treated for 24 h (t = 72 h) in the channels 1, 3 and 5. The drug concentrations in channel 1, 3 and 5 are 0, 3.36/105.15 and 6.72/210.10 μmol/L. After drug treatment, calcein-AM/PI were used to identify cell viability. Scale bar = 100 μm. **b** Normalized cell proliferation rate of single clones in representative microchambers after combined drug stimulation (t = 72 h). Drug concentrations of Ima/Res in channels 1−6 are 0, 1.69/52.68, 3.36/105.15, 5.04/157.63, 6.72/210.10 and 8/250 μmol/L respectively. **c** Normalized cell proliferation rate across the device under the three drug treatments. The maximum drug concentration in each treatment is indicated

Experimental deviation is recognized as inevitable in single-cell analysis due to the dynamic activities and susceptibilities of cells. Consequently, single K562 cell derived clones were also tested on our platform to minimize potential stochasticity in the DR analysis. As shown in Fig. 5a, single K562 cells were obtained and cultured in the device for 48 h to produce clones (Additional file 1: Fig. S3b), followed by the application of Ima, Res and Ima/Res for 24 h. The live/dead staining results indicated that fewer cells survived under the higher drug concentrations in all three treatments (Fig. 5a; Additional file 1: Figs. S7, S8), consistent with the findings of the single-cell based assays. Additionally, if we look closely at the images of t = 48 h and 72 h, it is clear that the first channel (channel 1) chambers show clear cell proliferation, and most of the third channel (channel 3) chambers maintained the number of cells, while the fifth channel (channel 5) chambers show few cells or increased amounts of cell debris. In addition, some cells also survived and showed very normal morphology even at high drug concentrations, suggesting that these were potentially drug-resistant cells [7]. Quantitative analysis of the proliferation rates in the representative microchambers after drug treatment confirmed these observations (Fig. 5b). In the case of single-drug treatments of Ima and Res, the results in Additional file 1: Figs. S7c and S8c were similar to those of the combined-drug group. To characterize the IC_50_ values, the averaged cell proliferation rate of the cells in the 6 channels was analyzed for the three groups of experiments (Fig. 5c). Table 1 shows the calculated IC_50_ values of 2.99, 90.03 and 1.58/49.38 µmol/L for Ima, Res, and Ima/Res treatment of the clones, respectively. Similar to the above single-cell based drug assays, Ima showed more specific inhibition of the K562 cell derived clones compared with Res, and the synergistic drug treatment was more efficient than the single treatment. Notably, all IC_50_ values of the clones were correspondingly higher than for the single cells. These results suggested that the single-cell clones were more resistant to drugs than single cells, suggesting that communication between cells might enhance their susceptibility to anticancer drugs [36].

### AML patient samples displayed significant heterogeneity in the single-cell based cytotoxicity assay

**Fig. 6:**
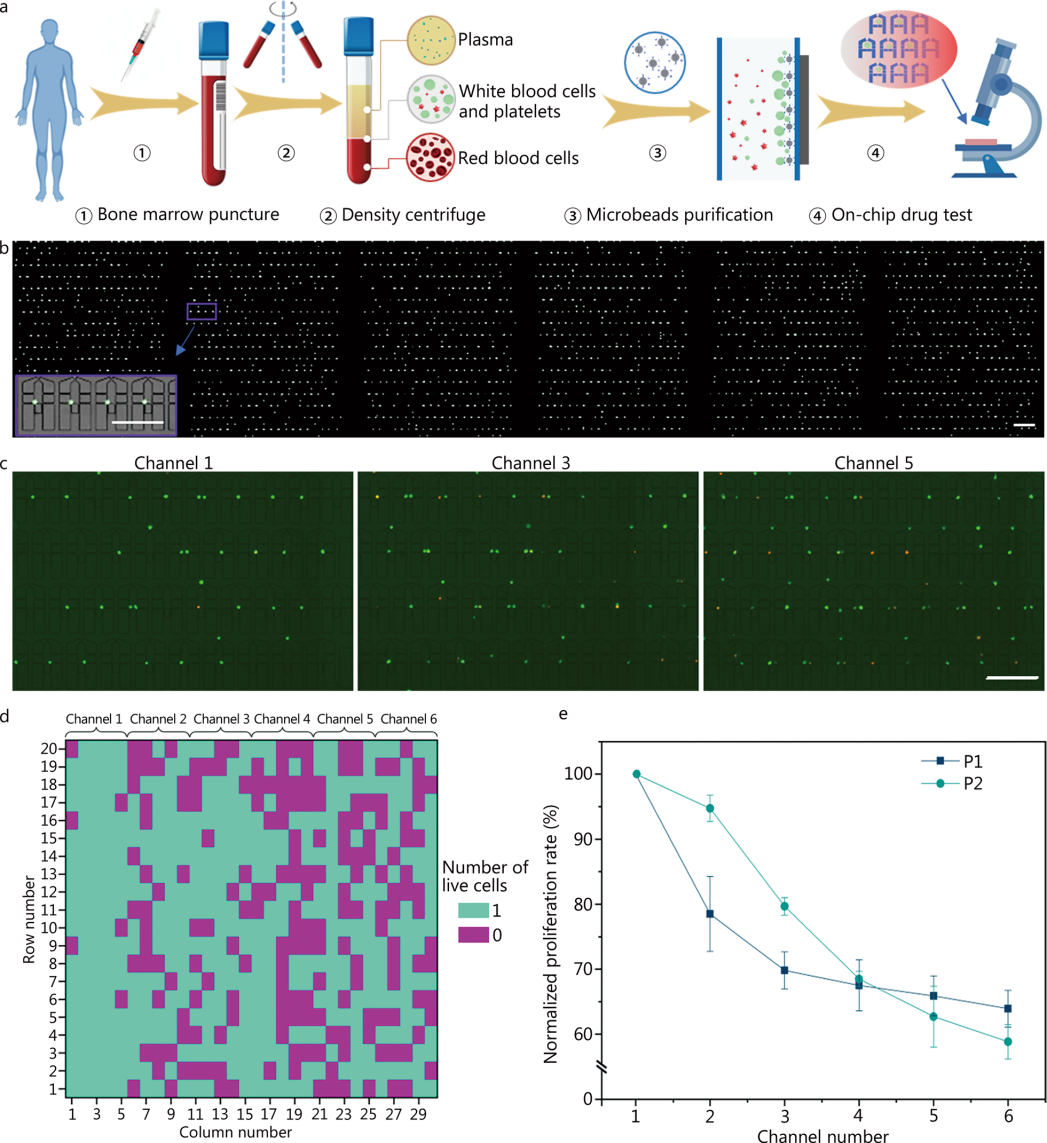
On-chip investigation of the drug (combination of Ara-C and DNR) effects on single cells isolated from AML patient bone marrow species. **a** Work flow showing the sample collection, centrifuge-based cell separation, anti-CD34 antibody labelled magnetic beads-based purification, and the on-chip assay. **b** High-throughput and high-efficiency capture of the primary cells with the modified MAC. The inset shows that the compacted microchamber perfectly matches the size of primary AML cells. The scale bar is 200 μm for **b** and 100 μm for the inset. **c** Representative microchambers in the channels 1, 3 and 5 showing the live/dead cells of patient sample 1 after Ara-C/DNR treatment for 24 h. The drug concentrations in channels 1, 3 and 5 are 0, 4.21/0.84 and 8.40/1.68 μmol/L, respectively. Scale bar = 100 μm. **d** Heatmap showing the live cells of patient sample 1 in the representative microchambers after drug treatment. The drug concentrations in channels 1 − 6 are 0, 2.11/0.42, 4.21/0.84, 6.31/1.26, 8.40/1.68 and 10.0/2.0 μmol/L for Ara-C/DNR, respectively. **e** Normalized cell proliferation rates of the two AML patient samples in the 6 channels with different drug concentrations. P1, P2 indicate patient number

Although most of our knowledge on tumors and tumor therapy originates from in vitro cell line models, there are, nevertheless, differences between cell lines and patient-derived tumor cells in terms of inherent cell heterogeneity and their associated microenvironments [37, 38]. Thus, cytotoxicity assays based on patient-derived tumor cells are more likely to recapitulate the actual conditions in clinical patients [39]. Moreover, to our knowledge, few reports have used massive parallel DR analysis for single patient-derived leukemic cells due to significant technical challenges in the handling of limited numbers of cells with much smaller sizes than the cell-line cells. Here, patient-derived AML cells were analyzed on our device to provide useful information for AML therapy (Fig. 6a). AML is the most common adult leukemia, and showed 20,240 new cases and 11,400 deaths in the USA alone in 2021, with a 5-year survival rate of only 29.5% [3]. Although some therapies have been developed, such as a combination of Ara-C and DNR as the standard of care, the problem of chemotherapy resistance remains unsolved [28].

CD34-positive cells were isolated from the bone marrow specimens of two AML patients (Additional file 1: Fig. S9a, b; Additional file 2: Table S1). Preliminary experiments were conducted to ensure cell viability before the on-chip analysis (Additional file 1: Fig. S9c). To efficiently capture these small primary leukemic cells, we carefully optimized the features of the microchamber and bypass (Additional file 1: Fig. S10a, b), resulting in a modified device with a throughput of approximately 5040 (840 × 6) capturing units. A typical patient-derived single-cell capturing image (Fig. 6b, Additional file 1: Fig. S10c) demonstrated the great capture efficiency of the modified MAC for handling primary cells. The concentration gradient formation and stability were also confirmed for this design (Additional file 1: Fig. S11a–c). After applying a combination of Ara-C and DNR for 24 h, the dose-dependent drug cytotoxicity was analyzed in the two patient-derived samples. This clearly showed the association between increased drug concentrations and the number of dead cells (Fig. 6c, Additional file 1: Fig. S11d). As with the K562 cells, drug-sensitive and drug-resistant cells were distinguished. The viability of single cells in the representative microchambers is shown in Fig. 6d, and the normalized cell proliferation rate of the two samples in the 6 channels is shown in Fig. 6e. The cell proliferation rate, measured by cell viability staining, was found to decrease in proportion to the increase in drug concentration. Nevertheless, the two response curves reflect the heterogeneity of patient-derived cells, which is further supported by the calculated IC_50_ values shown in Table 1 (1.75/0.35 µmol/L vs. 4.25/0.85 µmol/L). To be noted, it has been recognized that relapsed patients could demonstrate higher IC_50_ than that of those newly diagnosed. In our study, Patient 1 is a relapsed patient, and Patient 2 is a newly diagnosed patient. Our results displayed that Patient 1-derived cells had a lower IC_50_ than that of Patient 2-derived cells. This could be explained that the tested samples are too limited and the results are not statistically significant. Overall, this proof-of-concept study on patient samples demonstrated the significant potential of our platform, MAC, for single cell-based leukemia DR analysis.

## Discussion

DR-associated disease relapse is a main cause of leukemia treatment failure, although improvement has been made in reducing its overall death rate [4]. The advance of single-cell analysis techniques has opened a new dimension for profiling DR of tumor cells and shed light on tumor personal therapy [13, 17, 40]. Here, we developed an integrated microfluidic device for screening drug effects on massively arrayed leukemia single cells and single-cell derived clones, with expectation of establishing a general drug test platform for leukemia and other tumors. With our device, remarkable leukemia cell line cell and primary cell heterogeneity in terms of cell proliferation and drug susceptibility at the single cell level were successfully distinguished.

Since clinic-originated blood samples are always limited and precious, it thus represents a meaningful merit for single-cell device to have both a high-throughput and a high cell capture rate that do not waste the primary cells [7, 22]. Our mesh-based microchamber array balances the trapping unit throughput (as many as 4320 microchambers in a 2 cm^2^ footprint) and cell capture efficiency (as high as 80%), which allows the test of more than 3000 single cells in a single assay. Notably, parameters of the microchamber design can be adjusted, entailing flexibility of our device to accommodate to different cells and applications. Accordingly, the device demonstrated its capability to analyze single primary leukemia cells in a high-throughput way, which represents a significant challenge for current single-cell analysis platform due to the difficulty to handle the much smaller primary cells with high heterogeneity. Additionally, the sequential labels at selected positions greatly facilitate cell locating and continuous tracking. In terms of single-cell cloning, the house-like microchamber array is ideal for massive single-cell capture and culture to allow efficient single-cell derived clone formation. Although some reported microfluidic devices have achieved great single-cell capture efficiency, the on-site cell maintenance capability for high-throughput cell cloning is still technically challenging [22, 23]. Furthermore, the integration of an on-chip CGG greatly promotes our device as a simple but efficient tool for cancer drug effect analysis at the single cell level [13, 22, 41].

Due to the appropriate device design, no significant bias was observed across the microchamber array in cell capture, cell cloning and drug stimulation, which was proved by the consistency of cell performance in each column of the 6 channels. The drug-dose dependent effect on single cells and clones of the leukemia cell line and the primary cells demonstrated the robustness and minimal derivation of our microfluidic platform. The diverse IC_50_ values of single cells and clones upon drug treatment reflects the inherent difference of tested cell groups. Since the primary cell samples tested on our device are limited and the drug response difference of different patients, we obtained variant outcomes which can be improved by more tests of clinical samples. Overall, our device fulfills the expected goals to efficiently and dynamically characterize the drug susceptibility of single leukemia cells and clones.

Though we only demonstrated the application of screening anticancer drug effect by using the “SMART” microfluidic platform, we anticipate its wide use in single-cell based analysis. For example, it could be harnessed to profile the cytolytic activity of single T cells against the target cells in immunotherapy [9, 42], since our device allows great control and efficient tracking of small primary cells. Furthermore, it is possible to extract the cells of interest for downstream analysis (e.g., single cell sequence) if combining some automatic instruments such as laser capture microdissection systems. Thus another potential application of our strategy is to provide an efficient tool for single cell phenotypic tracking afore the subsequent single-cell sequencing, which allows the selection of certain interested cells for improving sequence depth [43].

Although our microfluidics-based platform has realized the above-mentioned benefits, improvements can be made to achieve better performance. For example, it could further increase the efficiency to introduce multiple drugs with multiple concentration gradients on the same chip. Moreover, high-throughput single cells analysis often generates enormous data of thousands of or even more cells (e.g., morphology change, proliferation rate, and cytotoxicity effect). Combination of automatic analysis approaches (e.g., artificial intelligence) will remarkably enhance the efficiency of microfluidics-based drug screening analysis [7].

## Conclusions

In this study, a new microfluidics-based strategy with SMART features was developed for leukemia single-cell heterogeneity and DR analysis. This was achieved by integrating a previously unidentified house-like microchamber array with a drug CGG. Our system has advantages in terms of throughput, cell capture efficiency, cell controlling precision, operation simplicity and function integration. These enable us to dynamically track thousands of single cells or clones over multiple time points upon drug treatment. This paves the way for efficiently evaluating the efficacy of cancer drugs on leukemia and also facilitates personalized therapeutic regimens for leukemia. More broadly, this strategy is beneficial to other single-cell based cell biology research.

## Supplementary Information

**Additional file 1: Fig. S1.** Design and simulation of the microfluidic device. **Fig. S2.** Overall pattern of the fabricated microfluidic device (a) and fabricated microchamber array (b). **Fig. S3.** Single cell array formed in the single-plexed device and the single-cell derived clones in a typical experiment. **Fig. S4.** Evaluation of on-chip cell proliferation. **Fig. S5.** Characterization of the on-chip concentration gradient generation. **Fig. S6.** Images of the single K562 cell array after the treatment of Imatinib (a) or Resveratrol (b) for 24 h on the microfluidic device. **Fig. S7.** Single drug treatment (Imatinib) of single K562 cell derived clones. **Fig. S8.** Single drug treatment (Resveratrol) to single K562 cell derived clones. **Fig. S9.** Separation of CD34+ acute myeloid leukemia cells from patient bone marrow species. **Fig. S10.** Optimized microchamber array for primary cell capture. **Fig. S11.** Evaluation of the concentration gradient generation on the modified device with fluorescein sodium (green) and sulforhodamine B (red) under 0.05 μl/min.

**Additional file 2: Table S1.** Acute myeloid leukemia patients’ characteristics.

## Data Availability

The data and materials used in the current study are all available from the corresponding author upon reasonable request.

## Abbreviations

AML: Acute myeloid leukemia
Ara-C: Cytarabine
CGG: Concentration gradient generator
CML: Chronic myeloid leukemia
CAD: Computer assistant design
CFD: Computational fluid dynamics
DR: Drug resistance
DNR: Daunorubicin
HTS: High-throughput screening
IC_50_: Half maximal inhibitory concentration
Ima: Imatinib
MAC: Microfluidic chamber Array and a six-Concentration gradient generator
PDMS: Polydimethylsiloxane
Res: Resveratrol
SMART: Simple, Massive and Multiplex, Alive, Retainable, and Trackable
