# Supplementary material for: An integrated microfluidics platform with high-throughput single-cell cloning array and concentration gradient generator for efficient cancer drug effect screening
Source: Mil Med Res. 2022 Sep 22;9:51. doi: 10.1186/s40779-022-00409-9 (PMC9494811; doi:10.1186/s40779-022-00409-9)
Supplement: Supplementary file 1 — Additional file 1: Fig. S1. Design and simulation of the microfluidic device. Fig. S2. Overall pattern of the fabricated microfluidic device (a) and fabricated microchamber array (b). Fig. S3. Single cell array formed in the single-plexed device and the single-cell derived clones in a typical experiment. Fig. S4. Evaluation of on-chip cell proliferation. Fig. S5. Characterization of the on-chip concentration gradient generation. Fig. S6. Images of the single K562 cell array after the treatment of Imatinib (a) or Resveratrol (b) for 24 h on the microfluidic device. Fig. S7. Single drug treatment (Imatinib) of single K562 cell derived clones. Fig. S8. Single drug treatment (Resveratrol) to single K562 cell derived clones. Fig. S9. Separation of CD34+ acute myeloid leukemia cells from patient bone marrow species. Fig. S10. Optimized microchamber array for primary cell capture. Fig. S11. Evaluation of the concentration gradient generation on the modified device with fluorescein sodium (green) and sulforhodamine B (red) under 0.05 μl/min. [file 40779_2022_409_MOESM1_ESM.pdf]

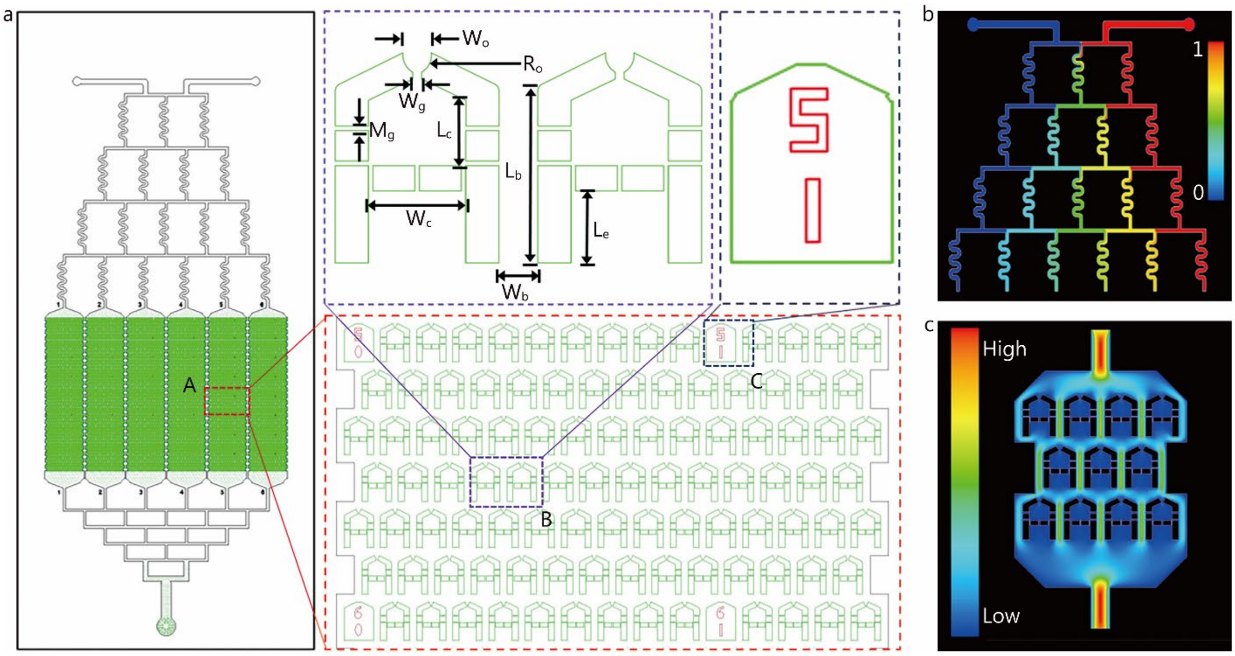

Fig. S1 Design and simulation of the microfluidic device. **a** Overall device designing. The enlarged view of A showed the arrayed microchambers. The enlarged view of B showed the detailed microstructure of the chamber, where the trap's top opening W_o is 16 μm, the trap's radius R_o is 12 μm, the trap's bottom gap W_g is 5.5 μm, the small gap M_g is 2.8 μm, the chamber interior length L_c is 40 μm, the chamber interior width W_c is 60 μm, the chamber exterior length L_b is 110 μm, the bypass width W_b is 24 μm, and the extended wall length L_e is 45 μm. The trap is for single cell capturing, the microchamber is for cell retention and long-term culture. The extended length L_e is to increase the flow resistance of the bypass. The enlarged view of C showed the marker for labelling the microchamber array. **b** Simulation result of the generated concentration gradients. **c** Simulation result of the flow velocity distribution in the microchamber array

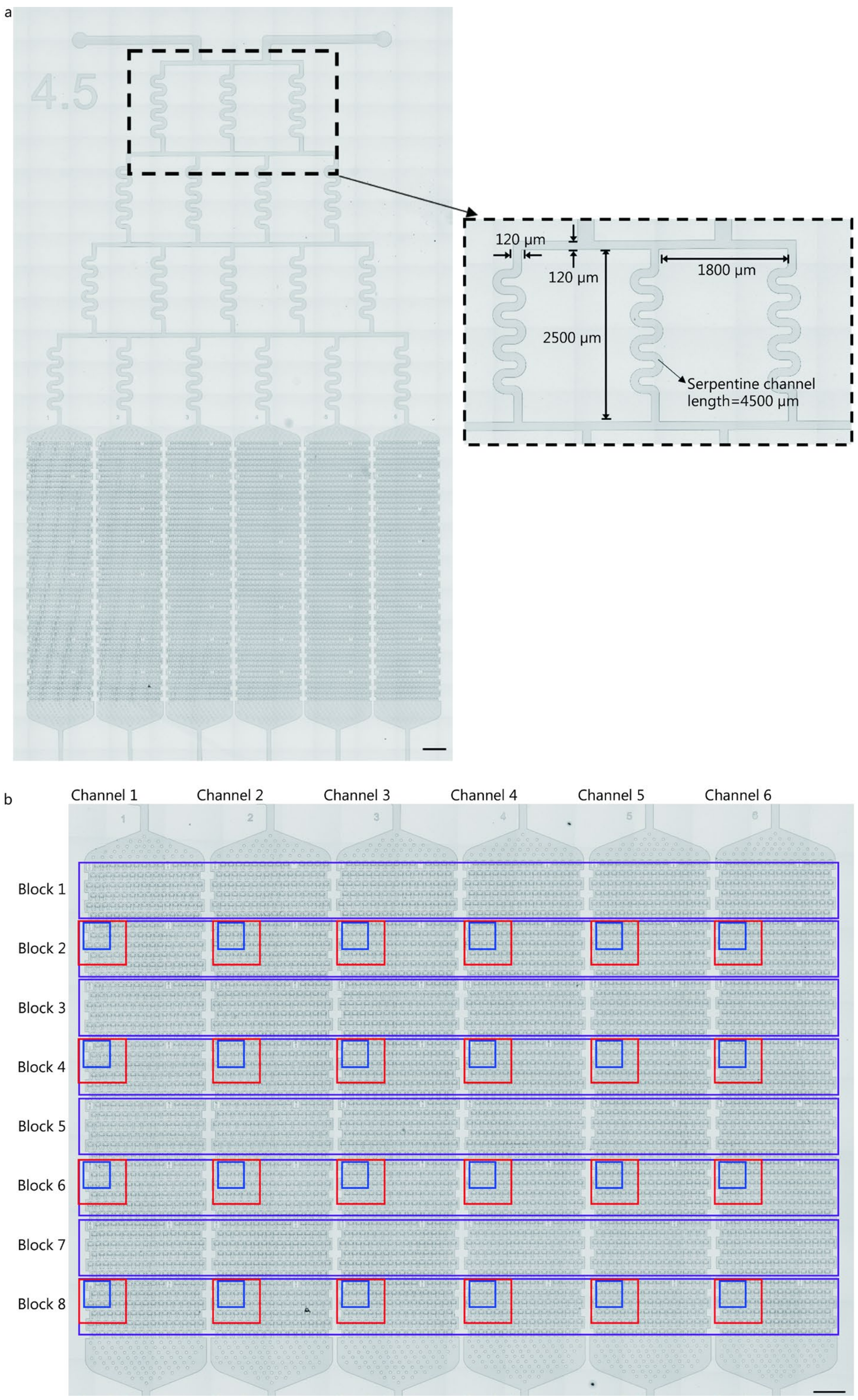

Fig. S2 Overall pattern of the fabricated microfluidic device (**a**) and fabricated microchamber array (**b**). The inset (in **a**) shows the detailed size of the microchannels. **b** There are 6 separate and identical channels, which is divided into 8 blocks (indicated by the purple rectangles) and each block contains 6 rows of microchambers. Sequential numbers were designed in the microchamber array for locating target microchambers. The red and blue rectangles indicate 5 × 5 and 3 × 3 chamber array respectively, which are used for single cell and single-cell derived clone analysis in relevant experiments. Scale bar = 500 μm

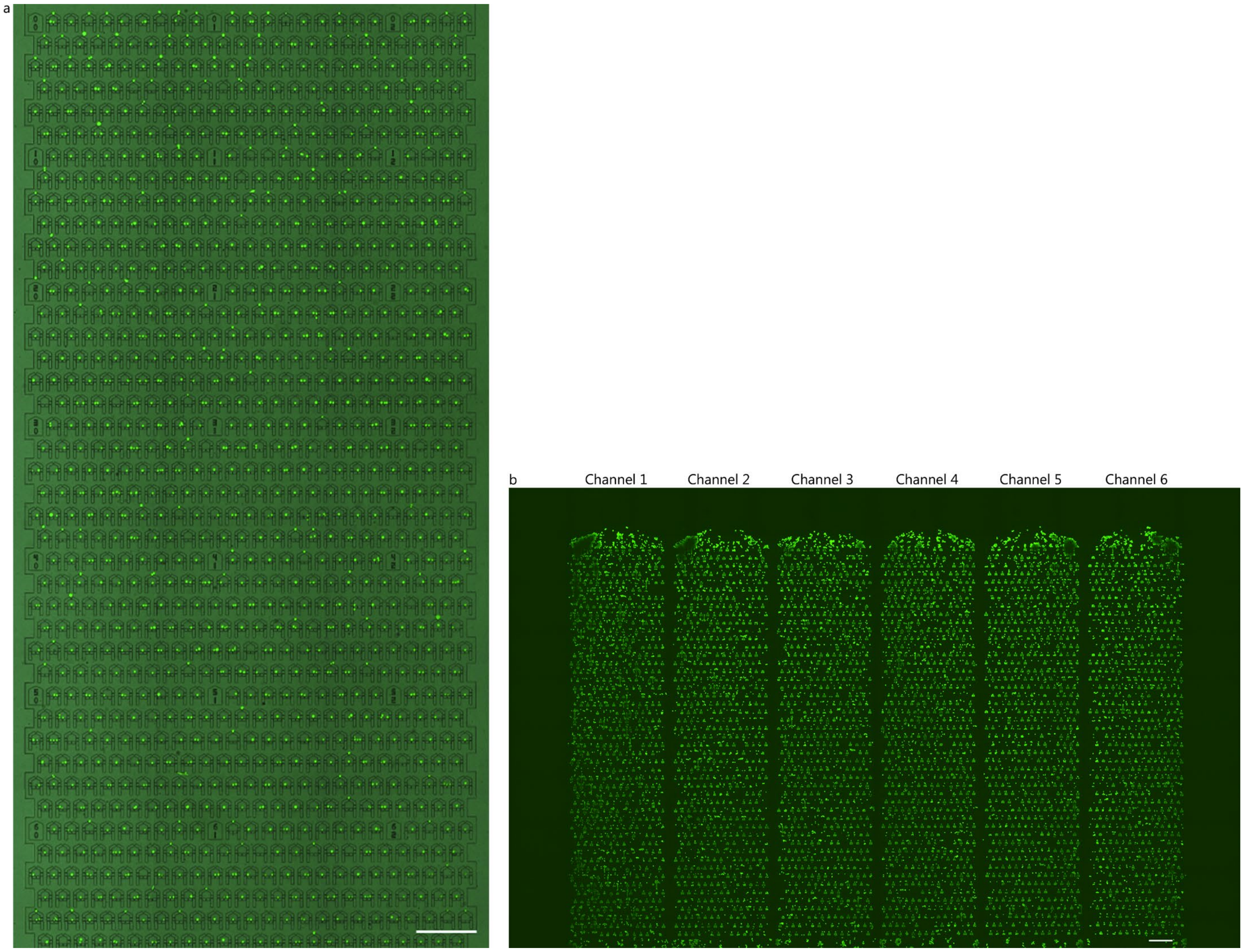

Fig. S3 Single cell array formed in the single-plexed device and the single-cell derived clones in a typical experiment. **a** Single cell array formed in the single-plexed device. The K562 cells were stained with calcein-AM. Scale bar = 300 µm. **b** A stitched image showing the single-cell derived clones in a typical experiment. The captured single cells were cultured in the device for 72 h. The result displayed that the clones are uniformly formed across the whole device. Scale bar = 500 µm

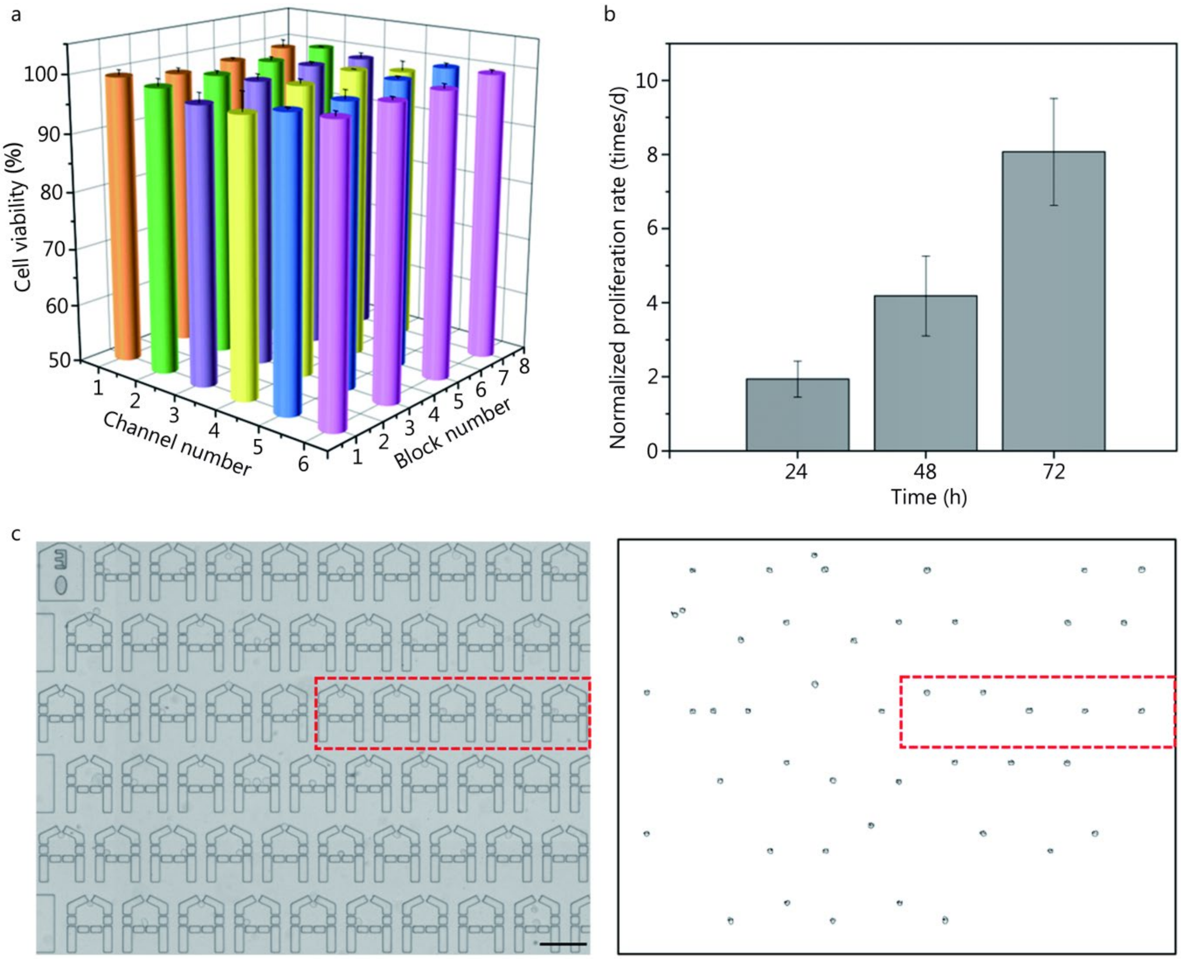

Fig. S4 Evaluation of on-chip cell proliferation. **a** Analysis of cell viability after the captured single K562 cells were cultured for 72 h. **b** Normalized proliferation rate at different time points. **c** ImageJ software was used to recognize cells captured in the device, and was further used to quantify cell proliferation rate vs. cell size. The red dotted rectangle highlights the chambers and the recognized cells. Scale bar = 100 μm

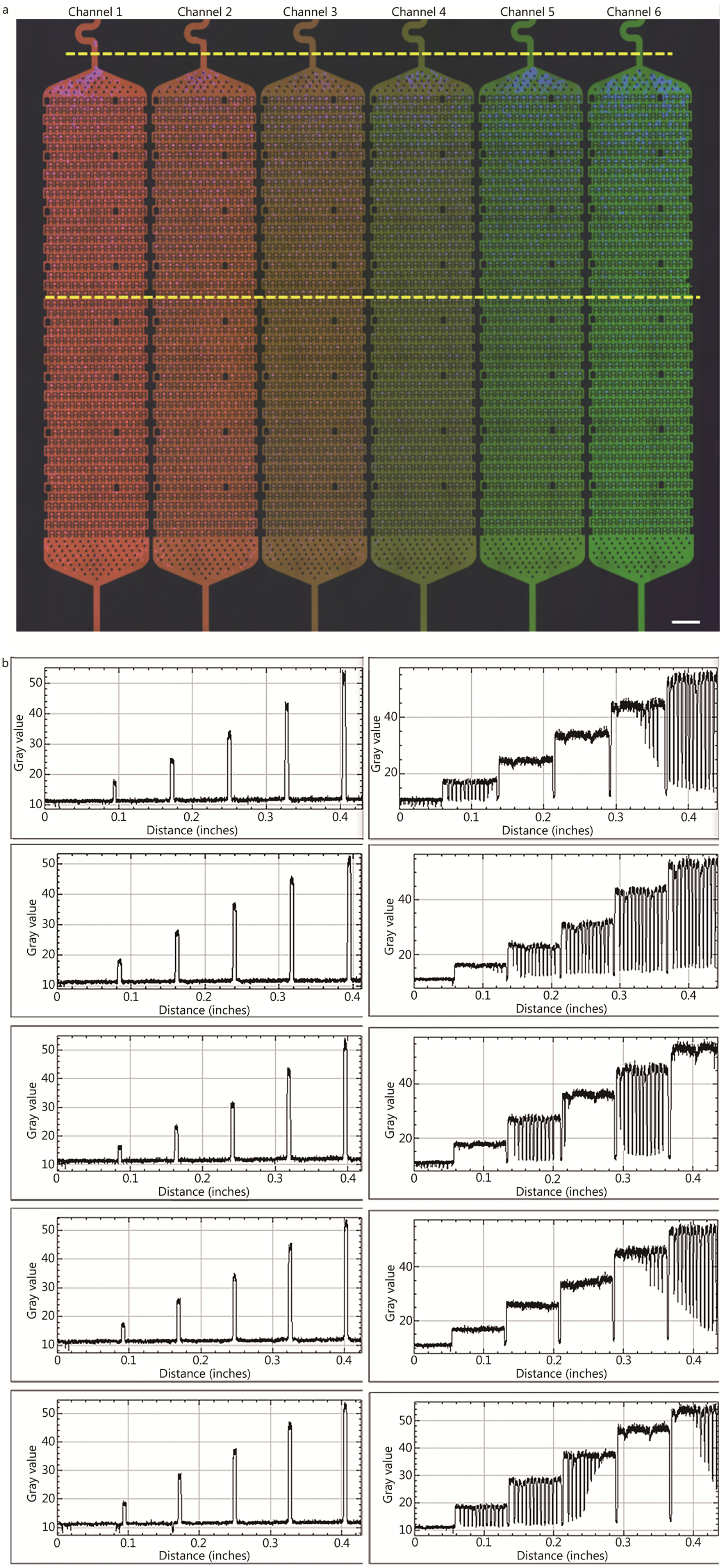
a
Channel 1
Channel 2
Channel 3
Channel 4
Channel 5
Channel 6
b
Gray value
Distance (inches)

Fig. S5 Characterization of the on-chip concentration gradient generation. **a** Fluorescein sodium (green) and sulforhodamine B (red) were injected, flowed through the Christmas-tree network and formed the gradients in the 6 channels. K562 cells were pre-cultured in the device to form an array of single clones (stained as blue). The two yellow dotted lines indicate the positions for concentration evaluation. The flow rate is 0.05 μl/min. Scale bar = 500 μm. **b** Stability evaluation of the concentration gradients. The green fluorescence intensities at the 6 channels (as indicated by the two yellow dotted lines in **a**) were extracted and plotted at different time points. These similar values at different time points displayed that the gradients are stable. Based on the measured fluorescent intensity data by ImageJ, concentration gradient profiles were normalized and fitted linearly, which can be expressed as: Y=0.2099X − 0.2091, R^2=0.9927, where Y is normalized fluorescent intensity or concentration coefficient, X is channel number (i.e., 1, 2, 3, 4, 5, 6), R^2 is linear coefficient. Further, concentration coefficient of each channel is 0, 0.21, 0.42, 0.63, 0.84 and 1.00, which is used for the drug concentration calculation in the drug-based assays

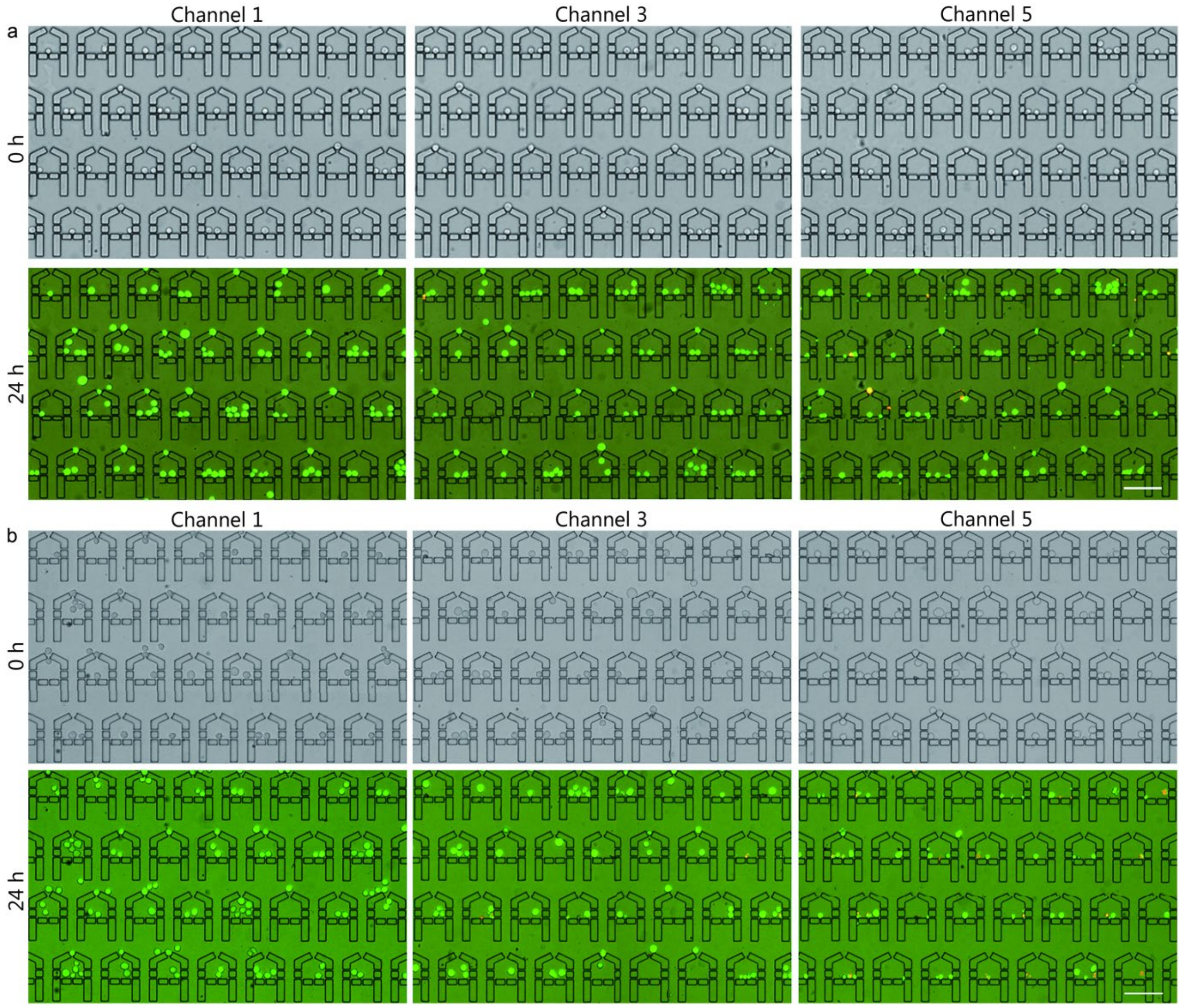

Fig. S6 Images of the single K562 cell array after the treatment of Imatinib (**a**) or Resveratrol (**b**) for 24 h on the microfluidic device. After drug treatment, calcein-AM/PI was used to identify cell viability. The drug concentrations in channels 1, 3 and 5 are 0, 1.26 and 2.52 μmol/L in **a**, and 0, 126.18, and 252.12 μmol/L in **b**, respectively. Scale bar = 100 μm

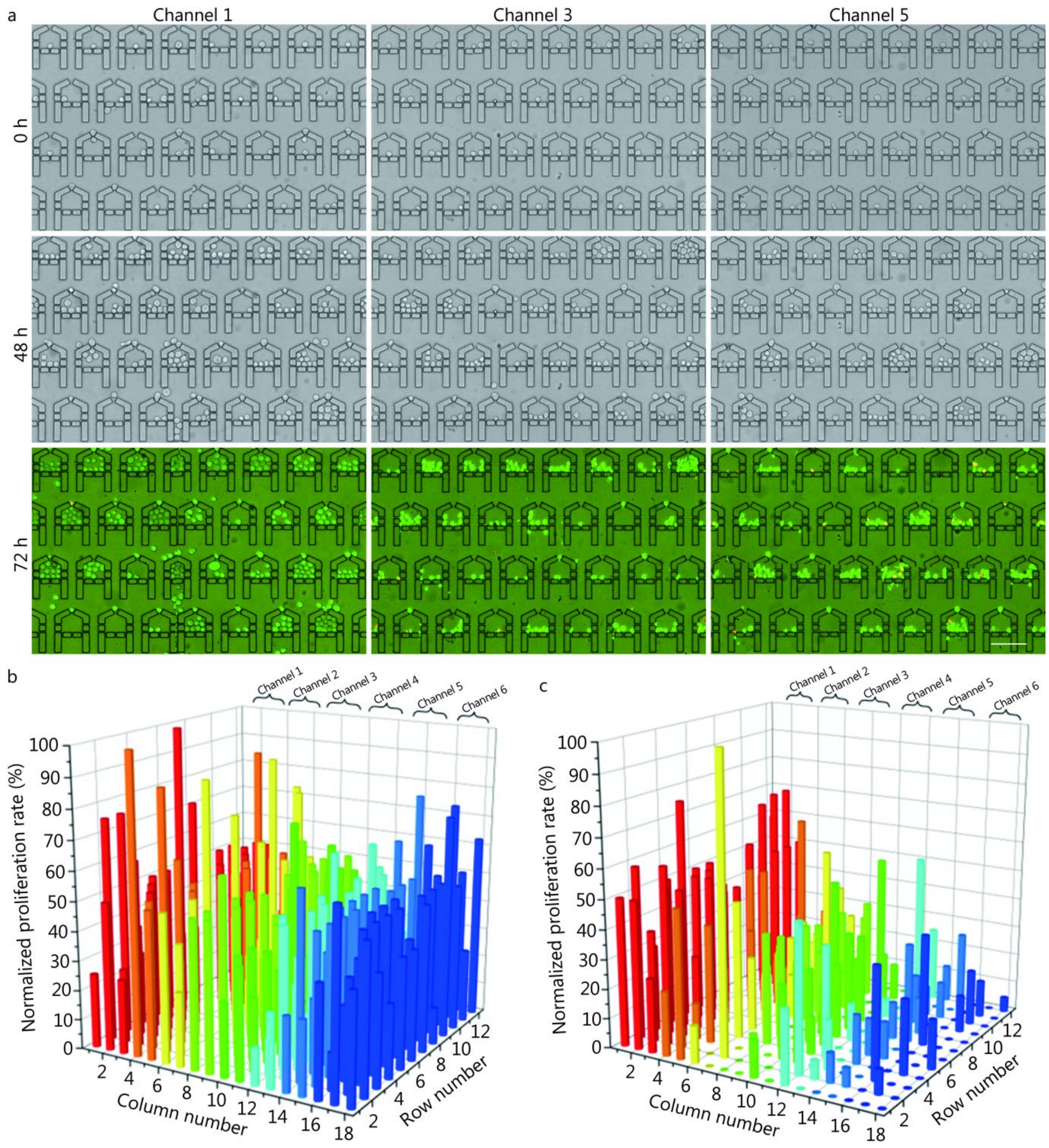

Fig. S7 Single drug treatment (Imatinib) of single K562 cell derived clones. Single cell array was generated on the device (t = 0 h) and cultured for 48 h, followed by drug treatment for 24 h (t = 72 h) on the microfluidic device. After drug treatment, calcein-AM/PI were used to identify cell viability. **a** Images of a representative area at different time points. The drug concentrations in channels 1, 3 and 5 are 0, 5.89 and 11.77 μmol/L. Scale bar = 100 μm. Proliferation rate across the device before (t = 48 h, **b**) and after (t = 72 h, **c**) drug treatment, respectively. The drug concentrations of channels 1 – 6 in **c** are 0, 2.95, 5.89, 8.83, 11.77 and 14.00 μmol/L, respectively. The results indicated that the cell growth is homogeneous before the drug treatment, but higher drug concentration significantly prohibited the cell growth

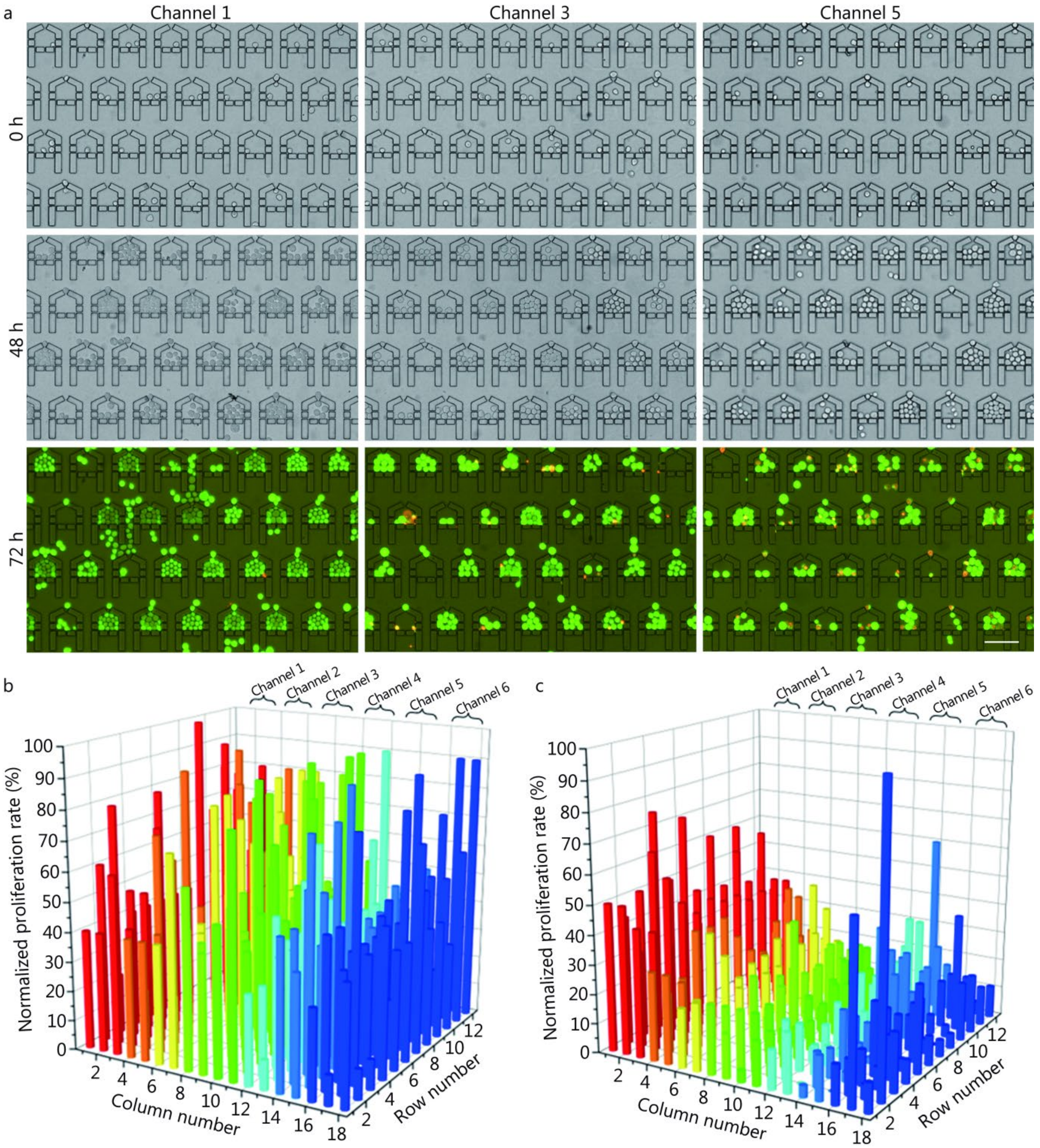

Fig. S8 Single drug treatment (Resveratrol) to single K562 cell derived clones. Single cell array was generated on the device (t = 0 h) and cultured for 48 h, followed by drug treatment for 24 h (t = 72 h) on the microfluidic device. After drug treatment, calcein-AM/PI were used to identify cell viability. **a** Images of a representative area at different time points. The drug concentrations in channels 1, 3 and 5 are 0, 210.30 and 420.20 μmol/L. Scale bar = 100 μm. Proliferation rate across the device before (t = 48 h, **b**) and after (t = 72 h, **c**) drug treatment, respectively. The drug concentrations of channels 1 – 6 in **c** are 0, 105.35, 210.30, 315.25, 420.20 and 500.00 μmol/L, respectively. The results indicated that the cell growth is homogeneous before the drug treatment, while higher drug concentration significantly prohibited the cell growth

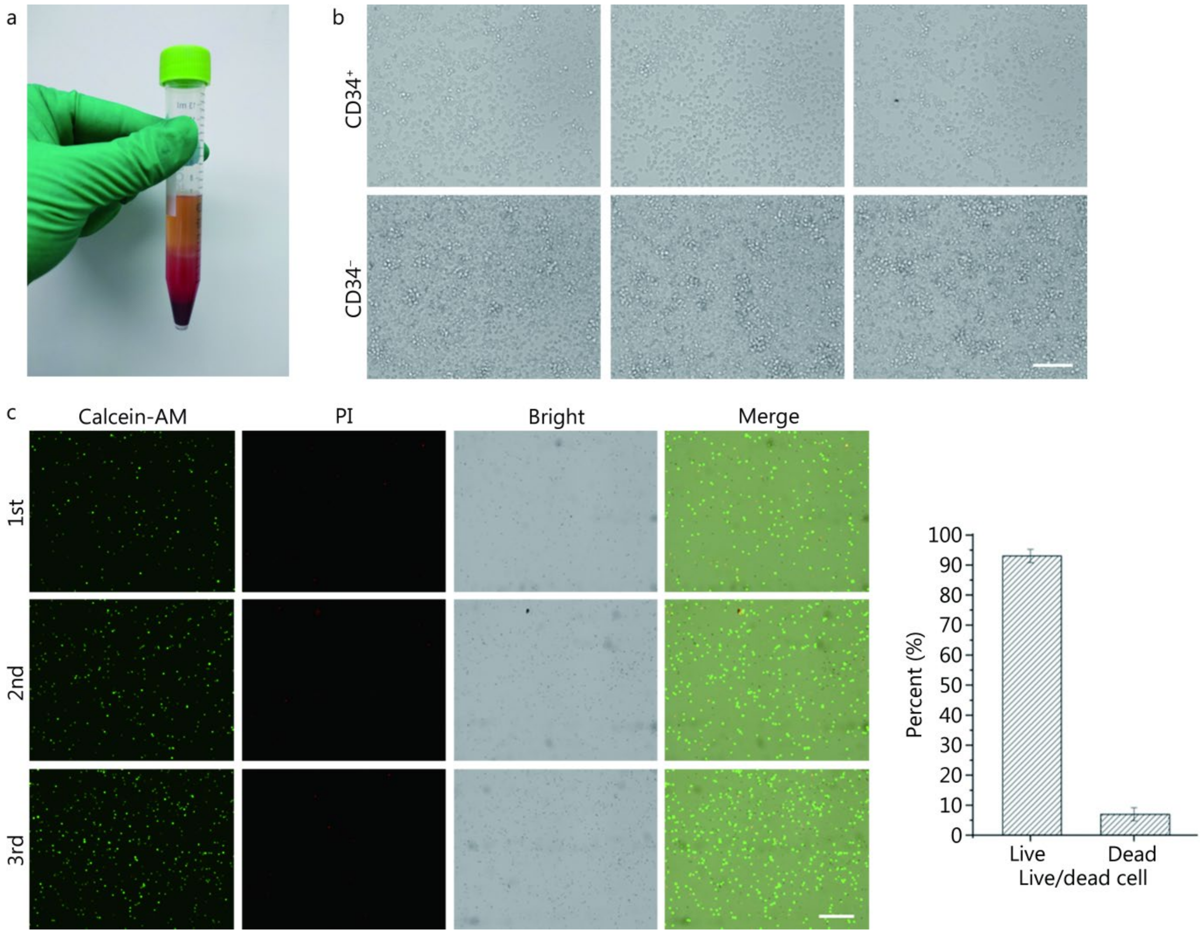

Fig. S9 Separation of $CD34^+$ acute myeloid leukemia cells from patient bone marrow species. **a** Density gradient centrifuge was first employed to isolate lymphocytes. **b** Images of the $CD34^+$ and $CD34^-$ cells. The images show that the two groups of cells have differences in morphology. The $CD34^+$ cells have relatively better monodispersity, which should benefit the cell loading and capturing. Microbeads conjugated antibody to CD34 was used to purify $CD34^+$ cells. **c** Cell viability of the $CD34^+$ cells was evaluated before loading into the microfluidic device using calcein-AM/PI. Three replicates were carried out. The staining of calcein-AM and PI were used to indicate the live and dead cells, respectively. Scale bar = 200 μm

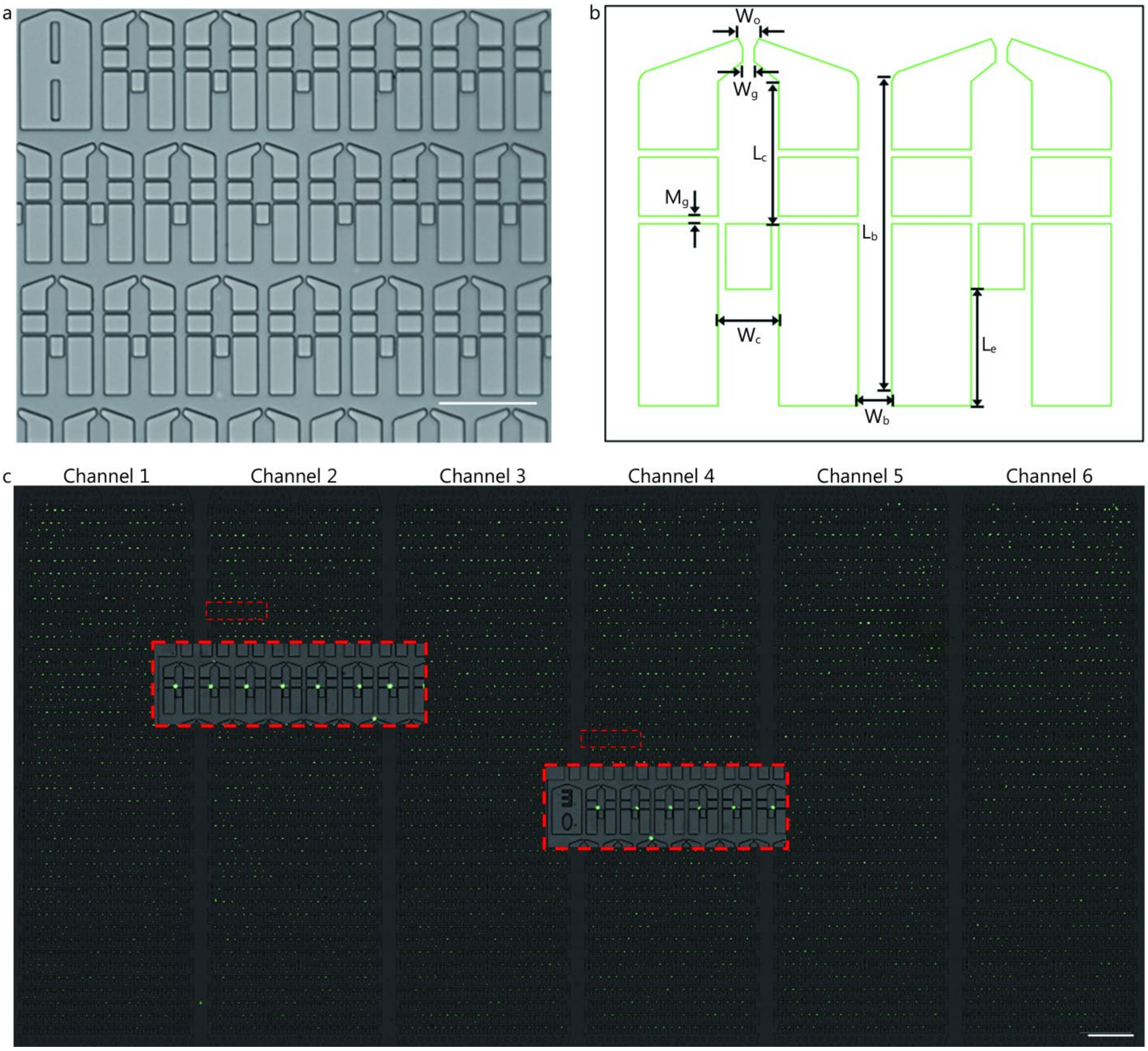

Fig. S10 Optimized microchamber array for primary cell capture. **a** Fabricated microchamber array under a 10 × objective. Scale bar = 100 µm. **b** Detailed size information of the microstructure. The trap's top opening W_o is 6.5 µm, the trap's bottom gap W_g is 3.8 µm, the small gap M_g is 2.5 µm, the chamber interior length L_c is 40 µm, the chamber interior width W_c is 20 µm, the chamber exterior length L_b is 110 µm, the bypass width W_b is 11 µm, and the extended wall length L_e is 38 µm. **c** A stitched image of the whole device showing the great performance of modified microchamber array for the capture of single primary AML cells. The enlarged view demonstrated that the microchamber matched well with the cell size. The cells were stained with calcein-AM. Scale bar = 500 µm. AML acute myeloid leukemia

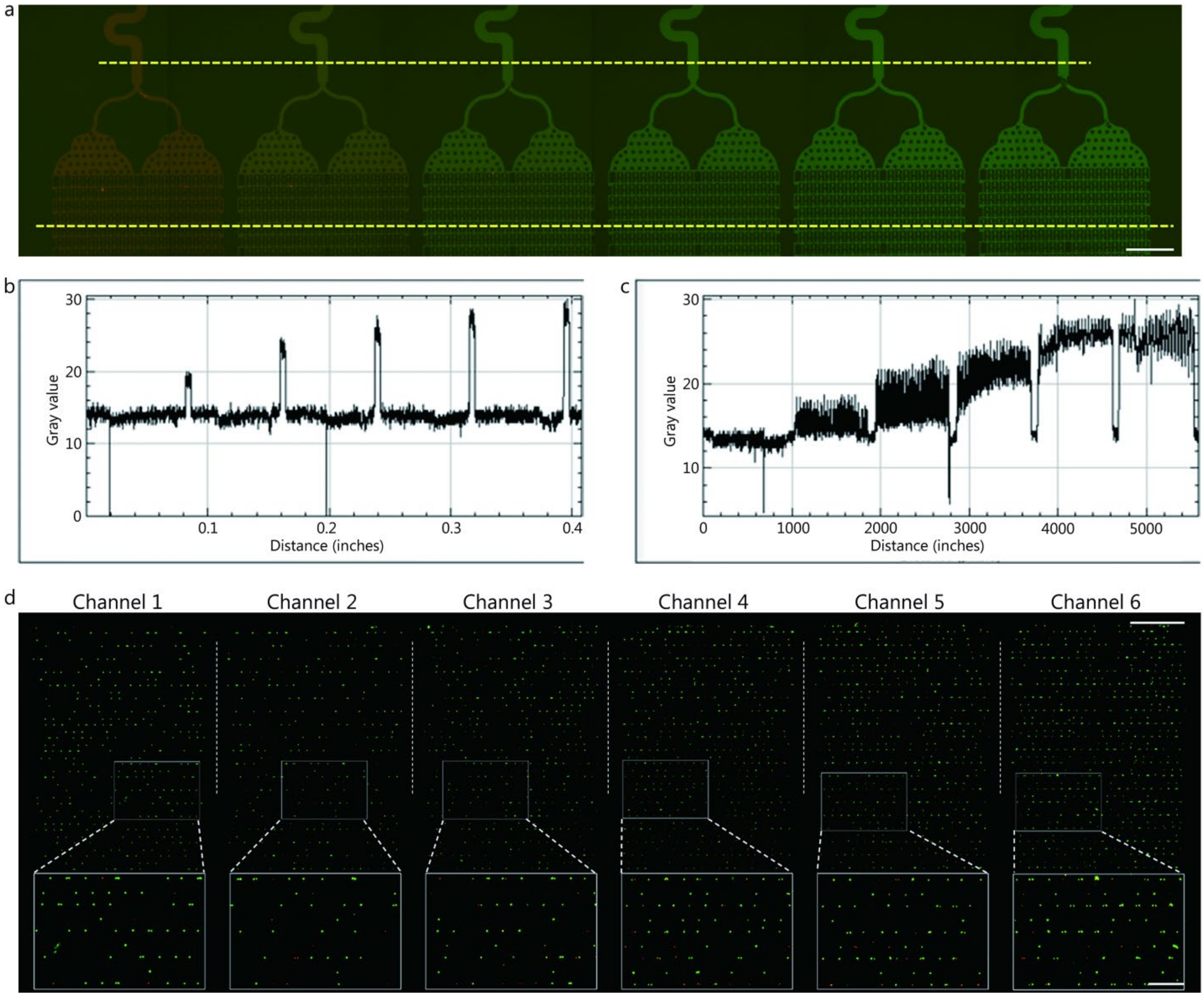

Fig. S11 Evaluation of the concentration gradient generation on the modified device with fluorescein sodium (green) and sulforhodamine B (red) under 0.05 μl/min. **a** A merged image showing the fluorescence distribution in the six channels of the device. The two yellow dotted lines indicate the positions for concentration evaluation. Scale bar = 500 μm. Fluorescence intensity quantified with ImageJ software at the position indicated by the top (**b**) and bottom (**c**) yellow dotted line in **a**. **d** Image showing the effect of combined drug treatment (cytarabine and daunorubicin) to patient sample 1. The cells of patient 1 were loaded into the device to form a single cell array, followed by drug treatment for 24 h. The drug concentrations in channels 1 − 6 are 0, 2.11/0.42, 4.21/0.84, 6.31/1.26, 8.40/1.68, 10.0/2.0 μmol/L for Ara-C/DNR, respectively. After that, calcein-AM/PI were used to identify cell viability. The insets demonstrated that more red spots appeared from the first channel to the sixth channel, which indicated higher drug concentration produced higher cytotoxicity to the primary cells. The scale bar is 500 μm for the stitched image and 300 μm for the insets
