# Supplementary material for: An integrated microfluidics platform with high-throughput single-cell cloning array and concentration gradient generator for efficient cancer drug effect screening
Source: Mil Med Res. 2022 Sep 22;9:51. doi: 10.1186/s40779-022-00409-9 (PMC9494811; doi:10.1186/s40779-022-00409-9)
Supplement: Supplementary file 2 — Additional file 2: Table S1. Acute myeloid leukemia patients’ characteristics. [file 40779_2022_409_MOESM2_ESM.pdf]

Table S1 Acute myeloid leukemia patients' characteristics

Patient number	Age (years)	Gender	BM volume (ml)	BM blasts (%)	AML disease phase
P1	50	Male	2.2	91	Relapse
P2	76	Female	1.0	89.7	Newly diagnosed

BM bone marrow, *AML* acute myeloid leukemia
